# Hypoxia-associated circPRDM4 promotes immune escape via HIF-1α regulation of PD-L1 in hepatocellular carcinoma

**DOI:** 10.1186/s40164-023-00378-2

**Published:** 2023-02-06

**Authors:** Zhi-Qiang Chen, Xue-Liang Zuo, Juan Cai, Yao Zhang, Guo-Yong Han, Long Zhang, Wen-Zhou Ding, Jin-Dao Wu, Xue-Hao Wang

**Affiliations:** 1 Hepatobiliary Center, The First Affiliated Hospital of Nanjing Medical University, Key Laboratory of Liver Transplantation, Chinese Academy of Medical Sciences, NHC Key Laboratory of Liver Transplantation, Nanjing, 210029 China; 2 Department of Gastrointestinal Surgery, The First Affiliated Hospital, Yijishan Hospital of Wannan Medical College, Wuhu, 241001 China; 3 Key Laboratory of Non-Coding RNA Transformation Research of Anhui Higher Education Institution, Wannan Medical College, Wuhu, 241001 China; 4 Department of Oncology, The First Affiliated Hospital, Yijishan Hospital of Wannan Medical College, Wuhu, 241001 China

**Keywords:** Circular RNA, Hepatocellular carcinoma, Hypoxia, Immune escape, PD-L1

## Abstract

**Background:**

Hypoxia is a hallmark of cancer, and is closely intertwined with tumor immune evasion. Circular RNAs (circRNAs) have been implicated in tumor response to immune checkpoint blockades. However, hypoxia-associated circRNAs that orchestrate the association between hypoxia and response to immunotherapy remain poorly understood. Here, we aimed to determine the roles of hypoxia-associated circRNAs in immune escape of hepatocellular carcinoma (HCC) cells.

**Methods:**

Differentially expressed hypoxia-associated circRNAs were determined using high-throughput sequencing technology. HCC patients treated with PD-1 blockade were enrolled to assess the clinical significance of circPRDM4. RT-qPCR, western blotting, flow cytometry, T cell-mediated tumor cell killing assay, and enzyme linked immunosorbent assay were used to investigate the roles of circPRDM4 in immune escape of HCC cells in vitro. Patient-derived xenograft mouse models and adoptive human tumor infiltrating lymphocyte-CD8^+^ T cell transfer were adopted to evaluate the effects of circPRDM4 in vivo. RNA pull-down, mass spectrometry, RNA immunoprecipitation, chromatin immunoprecipitation, chromatin isolation by RNA purification, dual-luciferase reporter assays, dot blotting, DNA in situ hybridization, and immunoprecipitation were utilized to examine the interaction between circPRDM4, HIF-1α, and *CD274* promoter.

**Results:**

We identified circPRDM4 as a hypoxia-associated circRNA in HCC. circPRDM4 was upregulated in responders to PD-1 blockade and associated with therapeutic efficacy. In vitro and in vivo experiments showed that circPRDM4 induced PD-L1 expression and promoted CD8^+^ T cell-mediated immune escape under hypoxic conditions. Mechanistically, circPRDM4 acted as a scaffold to recruit HIF-1α onto *CD274* promoter, and cemented their interaction, ultimately promoting the HIF-1α-mediated transactivation of PD-L1.

**Conclusions:**

These findings illustrated that circPRDM4 promoted immune escape of HCC cells by facilitating the recruitment of HIF-1α onto the promoter of *CD274* under hypoxia, thereby inhibiting CD8^+^ T cell infiltration in the tumor microenvironment. This work may provide a novel prognostic biomarker and therapeutic candidate for HCC immunotherapy.

**Supplementary Information:**

The online version contains supplementary material available at 10.1186/s40164-023-00378-2.

## Background

Hepatocellular carcinoma (HCC) represents the sixth most common cancer and the fourth leading cause of cancer-related death worldwide [1]. At least half of HCC patients are diagnosed at advanced stages, precluding potentially curative therapeutic approaches such as hepatectomy or liver transplantation. Immunotherapies that normalize immune responses in the tumor microenvironment have revolutionized the landscape of cancer treatment [2, 3]. Programmed death-ligand 1 (PD-L1) on tumor cells engages with programmed death 1 (PD-1) on immune cells, resulting in tumor immune evasion [4]. Therapeutic blockade of the PD-1/PD-L1 immune checkpoint pathways unleashes CD8^+^ T cell-mediated immune power against cancer and is a promising clinical anti-tumor therapeutic modality [5, 6]. In the treatment of advanced HCC, single-agent PD-1 blockade exhibits encouraging survival benefits in early-phase studies, however, the findings are not confirmed in phase III clinical trials [7, 8]. Hence, understanding the mechanisms underlying PD-1/PD-L1 dysregulation is necessary to improve efficacy of HCC immunotherapy.

Hypoxia is a common characteristic of tumors [9]. Cellular response to hypoxia has been implicated in several critical aspects of tumor progression, especially anti-cancer immunity [10]. Hypoxia affects tumor immunity and plays a crucial role in modulating the efficacy of anti-PD-1/PD-L1 treatment in cancers [11]. In hypoxic tumor cells, the central hypoxia-inducible factor HIF-1α transactivates *CD274*, the gene encoding PD-L1 protein, and leads to tumor immune escape from CD8^+^ cytotoxic T cells [12]. Hypoxia causes a HIF-1α-dependent upregulation of PD-L1 and suppresses T cell activation [13]. Therefore, investigating anti-tumor immunity under hypoxia may help to develop novel approaches for HCC immunotherapeutic strategy.

Circular RNAs (circRNAs) are covalent closed-loop structure without 5’ or 3’ ends and are resistant to RNA exonuclease, providing promising features to serve as potential biomarkers and therapeutic targets [14]. Fundamental regulatory functions of circRNAs have been demonstrated in various biological processes [15, 16]. Recent studies illustrate that dysregulation of circRNAs plays an essential role in PD-1/PD-L1-mediated anti-tumor immunity. The circIGF2BP3/PKP3 axis contributes to the immune escape of lung cancer cells through promoting the deubiquitination of PD-L1 [17]. circBART2.2 promotes the transcription of PD-L1 by binding RIG-I and causes subsequent immune evasion in nasopharyngeal carcinoma [18]. circDLG1 interacts with miR-141-3p and increases the expression of CXCL12, which promotes gastric cancer resistance to anti-PD-1-based therapy [19]. Nonetheless, the role of circRNA in HCC immune escape under hypoxic conditions remains obscure.

Herein, we demonstrated that hypoxia-associated circPRDM4 enhanced the immune escape of HCC cells under hypoxia. Mechanistically, circPRDM4 recruited HIF-1α onto the promoter of *CD274* and enhanced the HIF-1α-mediated transactivation of the *CD274* promoter. Subsequently, circPRDM4 increased tumoral PD-L1 expression level, inhibited CD8^+^ T cell infiltration, and contributed to immune evasion in HCC.

## Materials and methods

### Definition of normoxic and hypoxic conditions

Cells were cultured either under normoxic conditions (21% O_2_, 5% CO_2_, and 74% N_2_) or a hypoxic incubator (Memmert GmbH & Co. KG, Schwabach, Germany) providing a hypoxic environment (1% O_2_, 5% CO_2_, and 94% N_2_) for 24 h.

### circRNA sequencing

TRIzol reagent (Invitrogen, Carlsbad, CA, USA) was used to extract total RNA. RNA purity and integrity were examined using a NanoDrop ND-1000 spectrophotometer (Thermo Fisher Scientific, Waltham, CA, USA) and an Agilent 2200 TapeStation (Agilent Technologies, Santa Clara, CA, USA), respectively. rRNA was depleted using an Epicentre Ribo-Zero rRNA Removal Kit (Illumina, San Diego, CA, USA), and linear RNA was degraded using RNase R (Epicentre Technologies, Madison, WI, USA). The enrichment protocol following adaptor ligation was performed according to the instructions of the NEBNext Ultra RNA Library Prep Kit for Illumina (NEB, Beverly, MA, USA). The purified RNA was used for cDNA synthesis and sequencing. circRNAs were identified using both CIRCexplorer2 and CIRI2 pipelines. DESeq2 was adopted to determine the differentially expressed circRNAs (> twofold change and *P* < 0.05).

### Quantitative real-time PCR (RT-qPCR)

Total RNA was isolated using TRIzol reagent (Invitrogen). For RNase R treatment, total RNA was incubated with RNase R (3 U/μg; Epicentre Technologies) at 37℃ for 20 min before RT-qPCR detection. Reverse transcription was performed using the PrimeScript RT Master Mix (TaKaRa, Dalian, China). qPCR was performed using TB Green Premix Ex Taq (TaKaRa). The primers for RT-qPCR used in this study are listed in Additional file 1: Table S1. The 2^−ΔΔCt^ method was used to normalize the data.

### Immunofluorescence and fluorescence in situ hybridization (FISH)

Cells or tissue sections were fixed with 4% paraformaldehyde for 30 min, permeabilized with 0.5% Triton X-100 in PBS for 20 min, and then blocked with 5% bovine serum albumin for 1 h. After incubation with the indicated primary antibodies (CD8α, 1:400 dilution, Abcam, Waltham, MA, USA; HIF-1α, 1:400 dilution, Cell Signaling Technology, Danvers, MA, USA) at 4℃ overnight, the cells were washed three times by PBS at room temperature and incubated with the fluorescence-conjugated secondary antibodies for 30 min. Coverslips were mounted on slides using antifade mounting medium. Cell nuclei were stained with 4',6-diamidino-2-phenylindole (DAPI). Images were captured using a fluorescence microscope (Leica Microsystems, Mannheim, Germany).

FAM-labeled FISH probes specific to circPRDM4 (5’-TACACCCTGGCTTTGCGCACAAAC-3’) were designed and synthesized by GenePharma (Shanghai, China). FISH assays were performed using a Fluorescent In Situ Hybridization Kit (GenePharma) according to the manufacturer’s protocols. Briefly, cells were seeded on coverslips, fixed with 4% paraformaldehyde, and incubated with 0.5% Triton X-100 in PBS. After blocking, cells were incubated with FISH probes overnight and washed with SSC washing buffer. Cell nuclei were counterstained using DAPI. A confocal laser scanning microscope (PerkinElmer, Waltham, MA, USA) was used to capture the images.

### Nuclear and cytoplasmic fractionation

Subcellular fractionation of cell extracts was performed using the PARIS™ Kit (Invitrogen) following the manufacturer’s protocol. Briefly, the cells were washed using PBS, added with ice-cold cell fractionation buffer, and incubated on ice for 10 min. After the samples were centrifuged at 4℃ and 500 × g for 5 min, the supernatant containing the cytoplasmic fraction were collected for subsequent analyses. The pellet containing the nuclear fraction were added with cell disruption buffer and then collected for further experiments.

### Flow cytometry analysis of membrane PD-L1

The cells were centrifuged at 1000 × g for 5 min and collected. After incubating with the PE-conjugated PD-L1 antibody (1:200 dilution; BioLegend, San Diego, CA, USA) in dark for 30 min at 4 °C, cells were resuspended in FACS washing buffer on ice, and subjected to flow cytometry analyses. Samples were obtained and recorded in a FACS Aria II Cell Sorter (BD Biosciences, CA, USA), and data were analyzed with FlowJo software (TreeStar, Ashland, OR, USA).

### T cell-mediated tumor cell killing assay

T cells were activated using anti-CD3 (1 μg/ml) and anti-CD28 (5 μg/ml) antibodies (BD Biosciences, CA, USA) and human recombinant IL-2 (20 ng/ml) for 72 h. HCC cells with circPRDM4 knockdown or overexpression were seeded in plates overnight and incubated with activated T cells for 48 h. The cell culture media was removed. The adherent HCC cells were stained by crystal violent.

### Lactate dehydrogenase (LDH) release assay

LDH release assay was performed using a LDH Cytotoxicity Assay Kit (Beyotime, Shanghai, China) according to the manufacturer’s instructions. Briefly, HCC cells were plated in 24-well plates in a density of 4 × 10^4^ cells/well. Activated CD8^+^ T cells (2 × 10^5^ cells/well) were added at an effector-to-target ratio of 5:1 and cocultured for 16 h. The culture media were centrifuged at 400 × g for 5 min, and the supernatants were transferred into 96-well plates (120 μl/well) and incubated with LDH detection reagent (60 μl/well) for 30 min at room temperature in dark. Absorbance at 490 nm was detected using a microplate reader (Bio-Tek Elx 800; Bio-Tek Instruments, Winooski, VT, USA).

### Enzyme linked immunosorbent assay (ELISA)

To measure the levels of TNF-α and IFN-γ produced by cells, the supernatants were collected and the concentrations of TNF-α and IFN-γ were measured by the Human TNF-α ELISA Kit (MultiSciences, Hangzhou, China) and Human IFN-γ ELISA Kit (MultiSciences) according to the manufacturer’s guidelines. Optical densities were determined using a microplate reader (Bio-Tek Elx 800; Bio-Tek Instruments) at 450 nm.

### Animal experiments

For patient-derived xenograft (PDX) mouse model, 2 × 10^6^ patient-derived primary HCC cells mixed with Matrigel (Corning Inc., Corning, NY, USA) were subcutaneously transplanted in NCG mice. Human HCC tissues were minced into small pieces, treated with DNase I (20 μg/ml) and collagenase type II (100 U/ml in HBSS) for 30 min at 37 °C. Supernatants were collected and filtered through Falcon 70 μm Cell Strainer (Corning Inc.). The T cell fraction was collected using a prepared Percoll (GE Healthcare, Uppsala, Sweden) gradient by centrifuging at 800 × g for 30 min. Human CD8^+^ T cells were sorted from tumor infiltrating lymphocytes (TILs) by flow cytometer. CD8^+^ T cells were activated using anti-CD3 (1 μg/ml) and anti-CD28 (5 μg/ml) antibodies and human recombinant IL-2 (20 ng/ml) for 72 h. Each mouse was injected with 2 × 10^6^ of CD8^+^ T cells via caudal vein to reconstitute the human immune system. At day 7 after injecting with CD8^+^ T cells, circPRDM4 plasmid, empty vector plasmid, cholesterol-conjugated si-circPRDM4, or the negative control RNAi was intratumorally injected every three days for three weeks. Tumor growth was measured regularly. At day 28, mice were sacrificed and tumors were weighed and subjected for subsequent RT-qPCR and immunofluorescence analyses. To investigate whether knockdown or overexpression of circPRDM4 affect the proliferation of HCC cells under immunodeficient conditions, we also constructed the aforementioned PDX mouse models without adding immune cells to the mice. After intratumorally injection with circPRDM4 plasmid, empty vector plasmid, cholesterol-conjugated si-circPRDM4, or the negative control RNAi every three days for three weeks, the tumor growth and weight were compared between different groups.

Animal experiments were performed in compliance with the Animal Research: Reporting In Vivo Experiments (ARRIVE) guidelines and approved by the Institutional Animal Care and Use Committee of Nanjing Medical University (No. 10791) and the Animal Welfare and Ethical Committee of Wannan Medical College (No. LLSC-2020-090).

### RNA pull-down and mass spectrometry

RNA pull-down assay was performed using a Magnetic RNA–protein Pull-down Kit (Thermo Fisher Scientific). Briefly, cell lysates were incubated with biotin-labeled probes (RiboBio, Guangzhou, China) and streptavidin magnetic beads (Invitrogen). After incubation at 4℃ overnight, beads were separated magnetically and washed five times. For mass spectrometry analysis, the beads were incubated with ddH_2_O at 70℃ for 5 min. For western blotting, the beads were boiled in SDS for protein elution.

### RNA immunoprecipitation (RIP) assays

RIP assays were carried out using a Magna RIP RNA-Binding Protein Immunoprecipitation Kit (EMD Millipore, Billarica, MA, USA). Briefly, the cells were washed twice with ice-cold PBS, transferred to centrifuge tubes, and collected by centrifugation at 1500 rpm for 5 min at 4℃. Cells (1 × 10^7^) were resuspended in RIP Lysis Buffer and incubated on ice for 5 min. To confirm the binding between circPRDM4 and HIF-1α, 50 μl of beads coated with anti-HIF-1α antibody (5 μg; Cell Signaling Technology) or IgG antibody (5 μg; EMD Millipore) were incubated with the cell lysates in RIP Immunoprecipitation Buffer with rotating at 4℃ overnight. The protein-RNA complexes were washed six times with cold RIP Wash Buffer. The enriched RNA was purified and then isolated using TRIzol reagent and analyzed by RT-qPCR. To determine the HIF-1α domain required for circPRDM4 binding, cells were transfected with Flag-tagged bHLH (basic helix-loop-helix), PAS (Per-ARNT-Sim), or TAD (transactivation domain) truncated variants of HIF-1α, which were generated by GeneCreate (Wuhan, China). Cells were lysed and then incubated with anti-Flag antibody (5 μg; Cell Signaling Technology) or IgG antibody (5 μg; EMD Millipore) at 4 °C overnight. The abundance of protein-bound RNA was examined using RT-qPCR.

### Chromatin immunoprecipitation (ChIP) and chromatin isolation by RNA purification (ChIRP) assays

ChIP assays were performed using the Pierce Magnetic ChIP Kit (Thermo Fisher Scientific). Briefly, cells were crosslinked with 1% formaldehyde for 10 min at room temperature. Glycine was then added and incubated with the cells for 5 min to quench the crosslinking reaction. After washing twice with PBS, the cells were lysed in lysis buffer including protease and phosphatase inhibitors. Lysates were sonicated to shear DNA into smaller and workable pieces. Chromatin was incubated with anti-HIF-1α antibody (1:100 dilution; Cell Signaling Technology) or IgG antibody (2 μg; EMD Millipore) overnight at 4 °C. The following day, protein A/G magnetic beads were added and incubated for 2 h at 4 °C. After washing steps, DNA was eluted from beads and purified. The precipitated DNA was analyzed by qPCR or processed for ChIP-seq. *CD274* promoter-specific primers used for qPCR were as follows: *CD274* (− 2000 to − 1500 bp): Forward: 5’-ACACGAATCCTCACATTACT-3’; Reverse: 5’-AATCATATCCTCCTAGATGGC-3’; *CD274* (− 1500 to − 1000 bp):Forward: 5’-TTCGGGAACTTTGGGAAG-3’; Reverse: 5’-GCTGACACTGCCTTGATT-3’; *CD274* (− 1000 to − 500 bp):Forward: 5’-ATTATGACACCATCGTCTGT-3’; Reverse: 5’-TCGTGGATTCTGTGACTTC-3’; *CD274* (− 500 to 0 bp):Forward: 5’-CAGATGTTGGCTTGTTGTAA-3’; Reverse: 5’-GTATCTAGTGTTGGTGTCCTA-3’.

For ChIRP assays, 4 × 10^7^ cells were harvested and washed with PBS twice. Cells were fixed in 1% formaldehyde in PBS for 10 min at room temperature. Crosslinking was quenched with glycine for 5 min. After centrifuging at 1500 × g for 5 min at 4 ℃, cells were washed by ice-cold PBS twice. Crosslinked cell pellets were resuspended in lysis buffer and sonicated to produce chromatin fractions. circPRDM4 probe was designed at the junction site. All probes were synthesized with BiotinTEG at the 3’ end. Lysates were incubated with probes at 37 °C for 4 h. Streptavidin beads were then added to isolate probe binding complex. After washing steps, the bound DNA was quantified with RT-qPCR with *CD274* promoter-specific primers or processed for sequencing.

### Dual-luciferase reporter assay

The *CD274* wild-type or mutant promoter region was fused to the promoterless firefly luciferase gene of pGL3-Basic vector (Promega, Madison, MI, USA), and cells were transfected with luciferase reporter plasmid, the HIF-1α plasmid, the circPRDM4 plasmid, circPRDM4 truncated fragments, or the corresponding control plasmids. The luciferase activity of the cells was evaluated after 48 h using the Dual-Luciferase Reporter Assay System (Promega) according to the manufacturer’s instructions. The measurements were normalized to the ratio between firefly activity and renilla luciferase activity.

### Dot blotting

For dot blotting, wild-type or mutant circPRDM4 linearized RNAs were dropped onto Hybond-N^+^ membrane (Cytiva, Marlborough, MA, USA) followed by ultraviolet crosslinking. RNA signals were then detected using biotin-labeled single-stranded DNA segment of *CD274* promoter. Biotin signals were detected with HRP-conjugated streptavidin according to the manufacturer's instructions (Thermo Fisher Scientific).

### DNA in situ hybridization

The cells were treated with 0.1% NP-40 in 2 × SSC for 30 min, followed by digestion in proteinase K in TBS for 10 min at 37 °C. Serial dehydration was with ethanol to a final concentration of 100%. Cells were denatured for 8 min at 75 °C and replaced with ethanol to a final concentration of 100%. Afterwards, cells were air-dried and incubated with probes for *CD274* promoter and hybridization buffer overnight at 37 °C. The following day, cells were washed three times, 5 min each time: first with preheated 2 × SSC at 53 °C, then with preheated 2 × SSC with 0.1%NP-40 at 42 °C, and lastly with preheated 2 × SSC at 42 °C. Cell nuclei were stained with DAPI in dark for 10 min.

### Immunoprecipitation (IP)

For IP between HIF-1α and p300/CBP, cells were dissolved in RIPA, added with beads, mixed with anti-HIF-1α antibody (5 μg; Cell Signaling Technology) or IgG antibody (5 μg; EMD Millipore) at 4 °C overnight. The next day, the immunoprecipitated proteins were added with SDS-PAGE loading buffer, heated at 95 °C for 10 min, and subjected to western blotting. The dilution for anti-p300 antibody (Abcam) and anti-CBP antibody (Abcam) was 1:1000.

### Statistical analysis

Comparisons between groups were assessed using two-sided Student’s *t* test. Correlations between groups were evaluated using the Pearson’s correlation coefficient or Spearman’s ρ when appropriate. The significance of Kaplan–Meier survival curves was estimated with the log-rank test. All statistical analyses were performed using SPSS 26.0 (IBM Corporation, Armonk, NY, USA) or GraphPad Prism 9.0 (GraphPad Software, La Jolla, CA, USA), setting statistical significance at *P* < 0.05. *P < 0.05, **P < 0.01, ***P < 0.001, and “ns” stands for “no significance”.

Additional methodological details are included in Additional file 2: Supplementary Materials and Methods.

## Results

### circPRDM4 is a hypoxia-associated circRNA in HCC

**Fig. 1:**
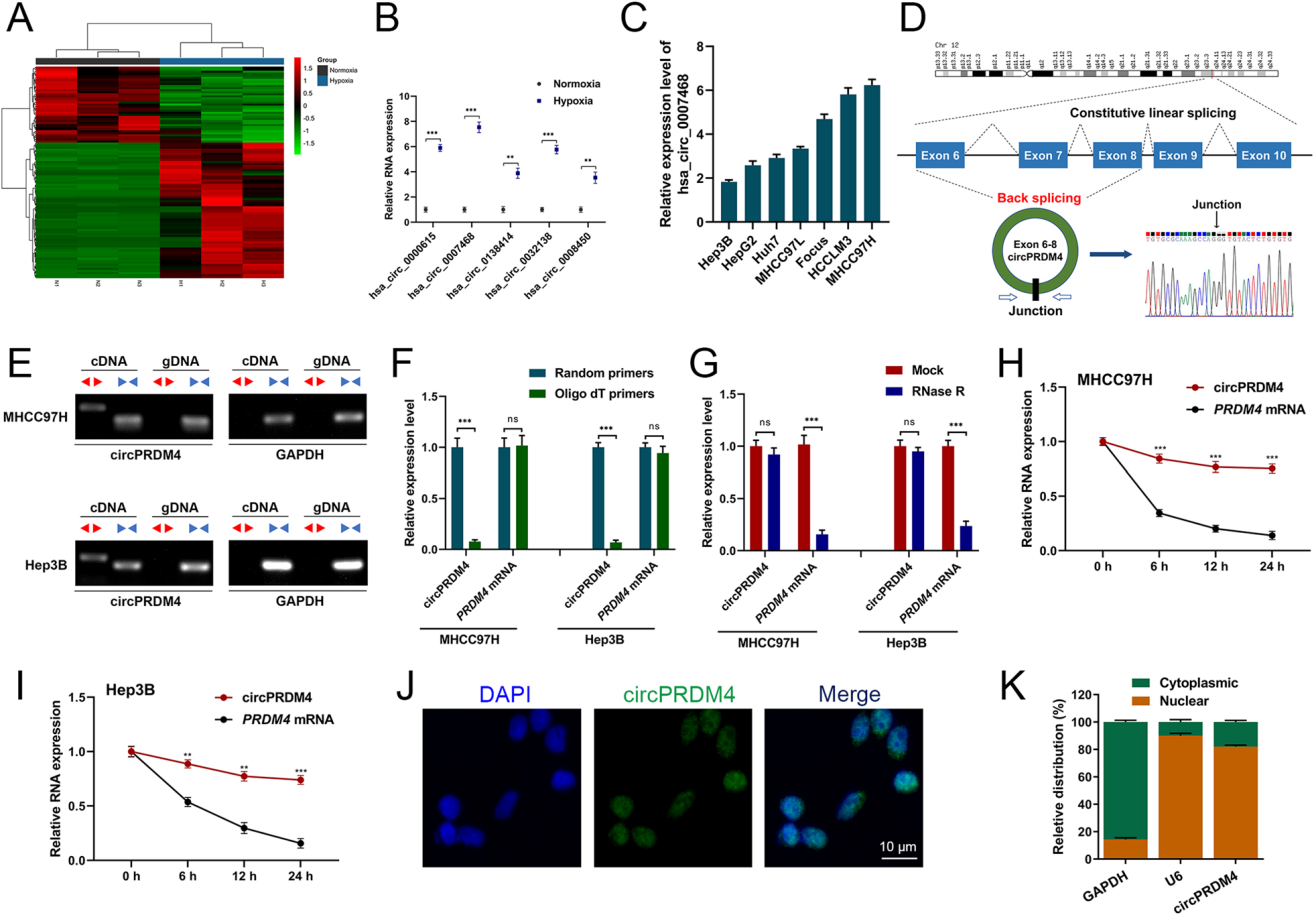
circPRDM4 is a hypoxia-associated circRNA in HCC.** A** Profiling of the differentially expressed circRNAs in HCCLM3 cells under hypoxia. **B** Expression levels of the five most regulated circRNAs were verified using RT-qPCR in HCCLM3 cells under hypoxic conditions. **C** Expression levels of hsa_circ_0007468 were examined in HCC cell lines. **D** Structure of circPRDM4. The back-splicing junction was confirmed by Sanger sequencing. **E** Agarose gel electrophoresis to check for the presence of circPRDM4. **F** RT-qPCR assays for circPRDM4 and *PRDM4* mRNA using the template cDNA reverse-transcribed by random primers and oligo dT primers. **G** Expression levels of circPRDM4 and *PRDM4* mRNA were evaluated in HCC cell lines treated with RNase R. **H** circPRDM4 and *PRDM4* mRNA levels were detected in MHCC97H cells after actinomycin D treatment. **I** circPRDM4 and *PRDM4* mRNA levels were detected in Hep3B cells after actinomycin D treatment. **J** FISH results showing that circPRDM4 was predominantly expressed in the nucleus of HCC cells. **K** Subcellular fractionation assays showing circPRDM4 was mainly observed in the nucleus of HCC cells. Data are shown as mean ± SEM. **, *P* < 0.01; ***, *P* < 0.001; ns, no significance

To identify hypoxia-associated circRNAs in HCC, we exposed HCC cell line HCCLM3 to normoxic or hypoxic treatment, and profiled the differentially expressed circRNAs. Compared to non-hypoxic controls, a total of 103 circRNAs with a > twofold change were identified in response to hypoxia (Fig. 1A). We selected the top five most regulated circRNAs for validation. Among these, hsa_circ_0007468 showed the greatest difference between hypoxic and normoxic HCCLM3 cells (Fig. 1B). The expression levels of hsa_circ_0007468 under hypoxic conditions were further assessed in several HCC cell lines. We observed high expression levels of hsa_circ_0007468 in MHCC97H and HCCLM3 cells, and low hsa_circ_0007468 levels in Hep3B and HepG2 cells (Fig. 1C), and these cell lines were chosen for subsequent experiments.

In silico analysis predicted that hsa_circ_0007468 was derived from exon 6 to exon 8 of the *PRDM4* gene. As demonstrated in Fig. 1D, Sanger sequencing verified the back-splicing junction of hsa_circ_0007468 (hereafter referred to as circPRDM4). To confirm the circular characteristics of circPRDM4, we designed divergent and convergent primers to amplify the back-spliced and linear transcripts, respectively. circPRDM4 can be amplified by divergent primers from cDNA but not gDNA (Fig. 1E). When random primers were replaced by oligo dT primers, the relative expression of circPRDM4, but not *PRDM4* mRNA, became barely detected (Fig. 1F). circPRDM4 was more resistant to RNase R degradation compared with the linear mRNA of *PRDM4* (Fig. 1G). After transcription inhibitor actinomycin D treatment, circPRDM4 was more stable than linear *PRDM4* mRNA (Fig. 1H, I). The circPRDM4 transcript was mainly observed in the nucleus of HCC cells using FISH and subcellular fractionation assays (Fig. 1J, K). Collectively, circPRDM4 is a hypoxia-associated circRNA in HCC.

### Upregulation of circPRDM4 is related to anti-PD-1 therapy response in HCC patients

Given that hypoxia is a key positive regulator of PD-L1 expression [12, 13], we set out to explore the role of hypoxia-associated circPRDM4 in HCC patients treated with PD-1 blockade. Twenty patients with advanced HCC treated with anti-PD-1 mAb were analyzed based on retrospective data. After 4 cycles of anti-PD-1 treatment, a responder is defined as having a partial response or a complete response, and a nonresponder is defined as having a progressive disease or stable disease, according to RECIST 1.1.

**Fig. 2:**
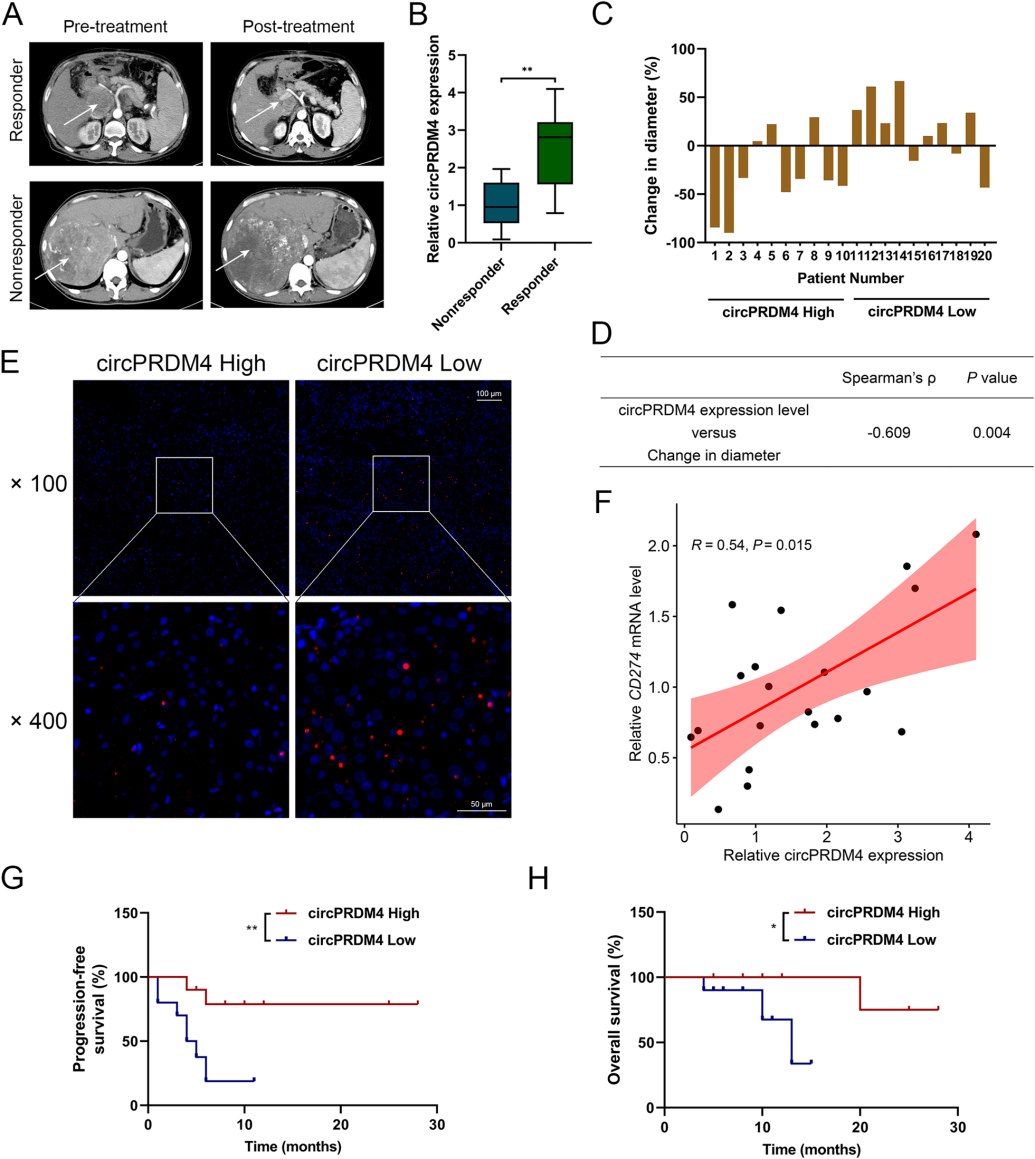
Upregulation of circPRDM4 is related to anti-PD-1 therapy response in HCC patients. **A** Representative radiographic results showing the tumor diameter change pre- and post-immunotherapy in responders and nonresponders. **B** Expression levels of circPRDM4 in tumor tissues from nonresponders and responders. **C** Difference in tumor diameter for all included patients. **D** Quantitative correlation between the change in tumor diameter and circPRDM4 expression level. **E** Representative immunofluorescence images showing CD8^+^ cells in tumors of the circPRDM4 high and circPRDM4 low groups. **F** Correlation analysis between circPRDM4 expression level and *CD274* mRNA level in HCC tissues. **G** Kaplan–Meier survival curves of the included patients’ PFS. **H** Kaplan–Meier survival curves of the included patients’ OS. Data are shown as mean ± SEM. *, *P* < 0.05; **, *P* < 0.01

Representative radiographic results are shown to annotate the tumor diameter change pre- and post-immunotherapy in responders and nonresponders (Fig. 2A). Increased expression level of circPRDM4 was found in tumor tissues from responding patients compared to those from nonresponders (Fig. 2B). The difference in tumor diameter for all included patients is demonstrated in Fig. 2C. There was a significant negative correlation between the relative increase of tumor diameter and circPRDM4 expression level (Fig. 2D). Compared to patients with lower circPRDM4 expression, decreased CD8^+^ cell infiltration was observed in patients with higher expression of circPRDM4 (Fig. 2E). Furthermore, we detected a positive correlation between circPRDM4 expression level and *CD274* mRNA level in HCC tissues (Fig. 2F). We observed prolonged progression-free survival (PFS) and overall survival (OS) in the group of anti-PD-1 mAb-treated HCC patients with high circPRDM4 expression (Fig. 2G, H). Together, circPRDM4 was upregulated in responders to PD-1 blockade and associated with therapeutic efficacy.

### circPRDM4 boosts PD-L1 expression and inhibits the CD8^+^ T cell-mediated anti-tumor immune response under hypoxic conditions

**Fig. 3:**
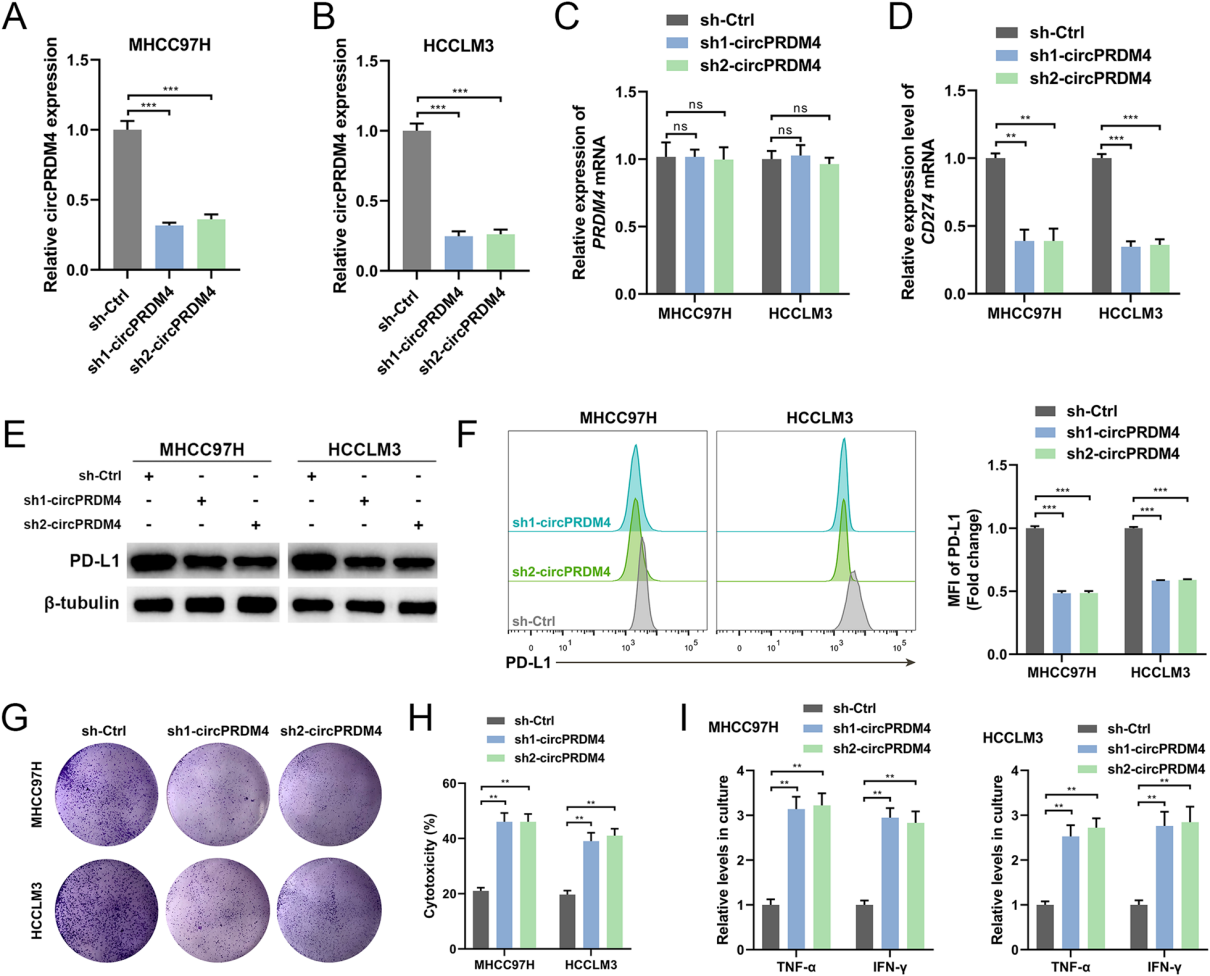
circPRDM4 knockdown suppresses PD-L1 expression and promotes the CD8^+^ T cell-mediated anti-tumor immune response under hypoxic conditions. **A** The knockdown efficiency of circPRDM4 in MHCC97H cells. **B** The knockdown efficiency of circPRDM4 in HCCLM3 cells. **C** Expression levels of *PRDM4* mRNA in circPRDM4-silenced MHCC97H and HCCLM3 cells. **D** Expression levels of *CD274* mRNA in circPRDM4-silenced HCC cells under hypoxia. **E** Expression levels of PD-L1 protein in circPRDM4-silenced HCC cells under hypoxia. **F** Cell surface PD-L1 expression levels in circPRDM4-silenced HCC cells under hypoxia. **G** T cell-mediated cancer cell-killing assay results of circPRDM4-silenced HCC cells. **H** LDH release assay results showing the cytotoxicity in circPRDM4-silenced HCC cells under hypoxia. **I** ELISA assays showing secreted TNF-α and IFN-γ from T cells cocultured with circPRDM4-silenced MHCC97H and HCCLM3 cells under hypoxia. Data are shown as mean ± SEM. **, *P* < 0.01; ***, *P* < 0.001; ns, no significance

To examine the functional role of circPRDM4 in immune escape of HCC cells under hypoxic conditions, we disrupted circPRDM4 by short hairpin-mediated RNA interference in two HCC cell lines with high circPRDM4 level. The knockdown efficiency of circPRDM4 in MHCC97H and HCCLM3 cells was validated by RT-qPCR (Fig. 3A, B). No statistical difference in *PRDM4* mRNA expression level was observed between HCC cells depleting circPRDM4 and the control cells (Fig. 3C). After hypoxia treatment, circPRDM4 depletion resulted in decreased expression levels of *CD274* mRNA and total PD-L1 protein (Fig. 3D, E). Additionally, the knockdown of circPRDM4 reduced cell surface PD-L1 expression in HCC cells exposed to hypoxia treatment (Fig. 3F). In T cell-mediated cancer cell-killing assay, downregulation of circPRDM4 in MHCC97H and HCCLM3 cells rendered the cells more vulnerable to lymphocytes (Fig. 3G). Additionally, we found that circPRDM4 knockdown had no significant effects on HCC cell proliferation in the absence of immune cells (Additional file 3: Fig. S1A). HCC cells transfected with circPRDM4 short hairpin RNAs (shRNAs) or control cells were incubated with activated CD8^+^ T cell under hypoxia, and the cytotoxicity was measured by LDH release assays. T cell cytotoxicity assays based on LDH release confirmed aggravated cytotoxicity in circPRDM4-silenced MHCC97H and HCCLM3 cells (Fig. 3H). After hypoxia treatment, circPRDM4 depletion alleviated tumor cell-mediated immune suppression, as determined by the elevated levels of secreted TNF-α and IFN-γ from cocultured T cells (Fig. 3I).

**Fig. 4:**
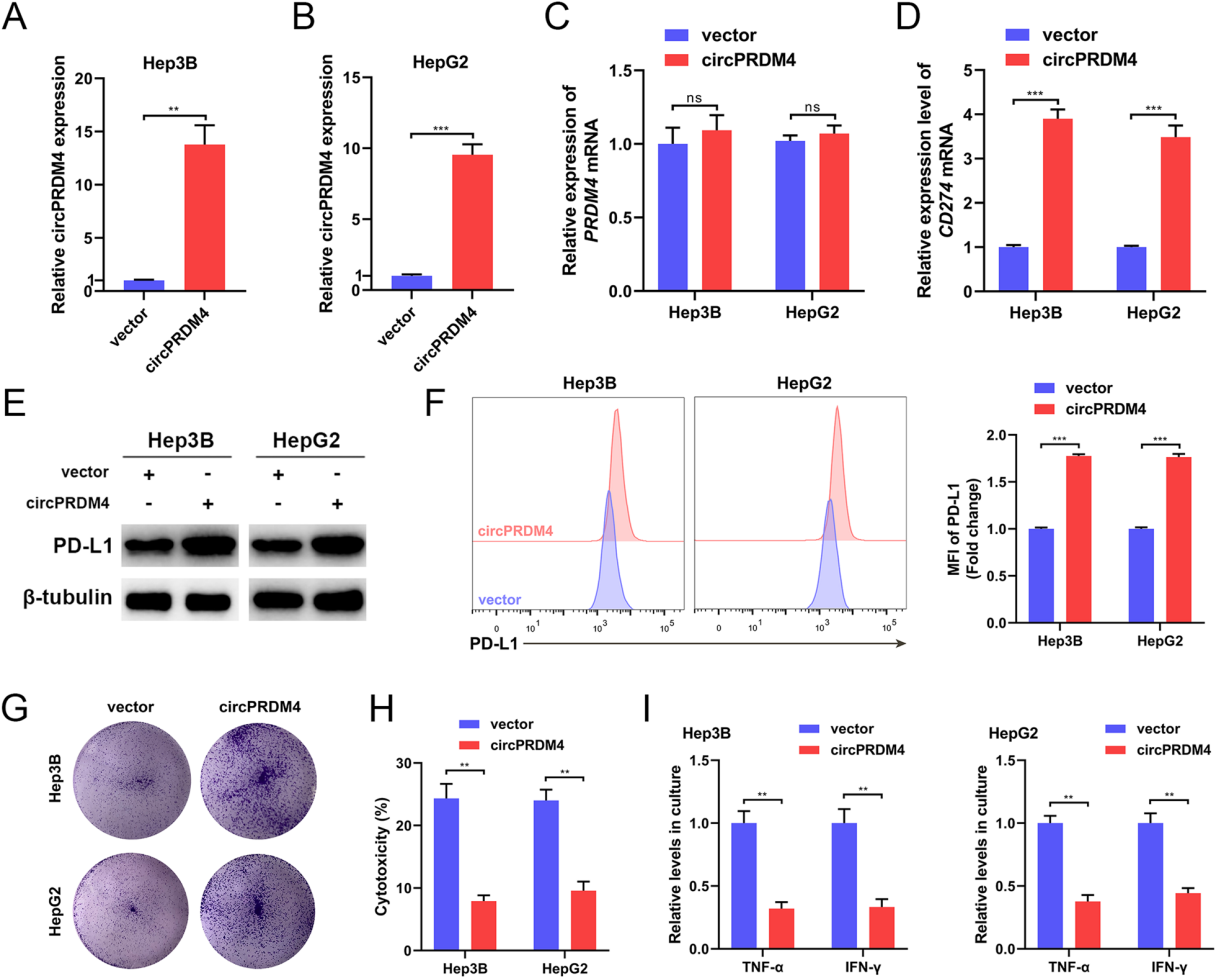
circPRDM4 overexpression boosts PD-L1 expression and inhibits the CD8^+^ T cell-mediated anti-tumor immune response under hypoxic conditions. **A** The overexpression efficiency of circPRDM4 in Hep3B cells. **B** The overexpression efficiency of circPRDM4 in HepG2 cells. **C** Expression levels of *PRDM4* mRNA in circPRDM4-overexpressing Hep3B and HepG2 cells. **D** Expression levels of *CD274* mRNA in circPRDM4-overexpressing HCC cells under hypoxia. **E** Expression levels of PD-L1 protein in circPRDM4-overexpressing HCC cells under hypoxia. **F** Cell surface PD-L1 expression levels in circPRDM4-overexpressing HCC cells under hypoxia. **G** T cell-mediated cancer cell-killing assay results of circPRDM4-overexpressing HCC cells. **H** LDH release assay results showing the cytotoxicity in circPRDM4-overexpressing HCC cells under hypoxia. **I** ELISA assays showing secreted TNF-α and IFN-γ from T cells cocultured with circPRDM4-overexpressing Hep3B and HepG2 cells under hypoxia. Data are shown as mean ± SEM. **, *P* < 0.01; ***, *P* < 0.001; ns, no significance

HCC cell lines stably overexpressing circPRDM4 were successfully constructed (Fig. 4A, B). circPRDM4 overexpression did not change the expression of *PRDM4* mRNA (Fig. 4C). Under hypoxic conditions, expression levels of *CD274* mRNA, total PD-L1 protein, and cell surface PD-L1 were augmented in Hep3B and HepG2 cells overexpressing circPRDM4 (Fig. 4D–F). Upregulation of circPRDM4 significantly hindered T cell-induced cell death according to the results from T cell-mediated cancer cell-killing assays (Fig. 4G). Moreover, the effects on HCC cell proliferation after circPRDM4 overexpression were comparable in the absence of immune cells (Additional file 3: Fig. S1B), suggesting that circPRDM4 mainly functioned through regulating anti-tumor immune response. LDH release assays after hypoxia treatment showed that tumor cell death induced by T cells were thwarted after circPRDM4 upregulation (Fig. 4H). circPRDM4 overexpression compromised the secretion of TNF-α and IFN-γ in response to hypoxia (Fig. 4I). Collectively, these results suggested that circPRDM4 induced PD-L1 expression and promoted CD8^+^ T cell-mediated immune escape under hypoxic conditions.

### circPRDM4 facilitates tumor growth and immune escape in vivo

**Fig. 5:**
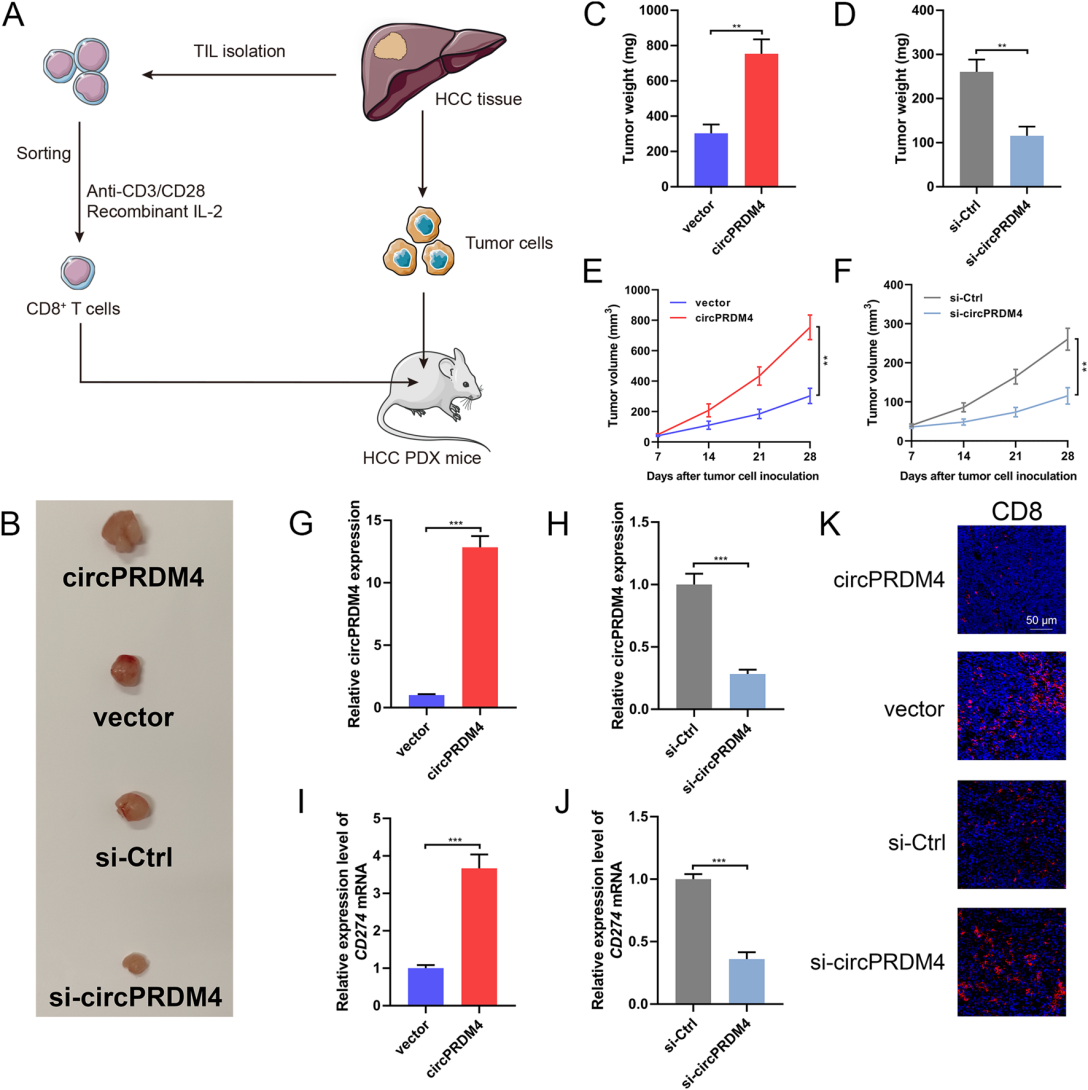
circPRDM4 facilitates tumor growth and immune escape in vivo. **A** Illustration of the construction of the PDX mouse model and adoptive human TIL-CD8^+^ T cell transfer. **B** Representative tumor images of each group of PDX mice at the end of treatment. **C** Tumor weights of the xenografts with circPRDM4 overexpression at the end of treatment. **D** Tumor weights of the xenografts with circPRDM4 knockdown at the end of treatment.** E** Tumor growth curves of the xenografts with circPRDM4 overexpression. **F** Tumor growth curves of the xenografts with circPRDM4 knockdown.** G** Expression levels of circPRDM4 in xenografts with circPRDM4 overexpression. **H** Expression levels of circPRDM4 in xenografts with circPRDM4 knockdown.** I** Expression levels of *CD274* in xenografts with circPRDM4 overexpression. **J** Expression levels of *CD274* in xenografts with circPRDM4 knockdown. **K** Representative immunofluorescence images showing infiltrated CD8^+^ cells in each group of xenografts. Data are shown as mean ± SEM. **, *P* < 0.01; ***, *P* < 0.001

To test the effects of circPRDM4 on HCC growth and immune evasion in vivo, we constructed a humanized mouse model as illustrated in Fig. 5A. Tumor infiltrating CD8^+^ T cells from HCC patients were subsequently adoptively transferred into immune-compromised NCG mice harboring HCC PDX. We observed that circPRDM4 overexpression markedly promoted tumor volume and weight, and knockdown of circPRDM4 significantly inhibited the growth and weight of tumors (Fig. 5B–F). The overexpression and knockdown efficiencies of circPRDM4 in tumors were verified using RT-qPCR (Fig. 5G, H). Additionally, in vivo experiments confirmed that circPRDM4 overexpression could increase *CD274* expression levels, whereas knockdown of circPRDM4 led to a reduced expression level of *CD274* (Fig. 5I, J). CD8 immunofluorescence staining of the tumors showed fewer CD8^+^ cells in the group of circPRDM4-overexpressing tumors and more CD8^+^ cells in the circPRDM4-silenced group, as compared to the corresponding controls (Fig. 5K). We further performed the animal experiments without adding immune cells to the mice. As shown in Additional file 3: Fig. S1C–I, we found that circPRDM4 knockdown or overexpression did not exert significant effects on HCC growth in vivo. Taken together, these data indicated that circPRDM4 promoted HCC growth and facilitated immune escape in vivo.

### circPRDM4 binds to HIF-1α under hypoxia

Given that circPRDM4 was mainly located in the nucleus, we wondered whether circPRDM4 could regulate the expression of its host gene. Results from RT-qPCR and western blotting assays showed that circPRDM4 had no influence on the expression of its host gene (Figs. 3C, 4C and Additional file 4: Fig. S2A). Furthermore, no protein-coding sequence could be found in circPRDM4 according to results from CPAT and circRNADb databases (Additional file 4: Fig. S2B, C).

**Fig. 6:**
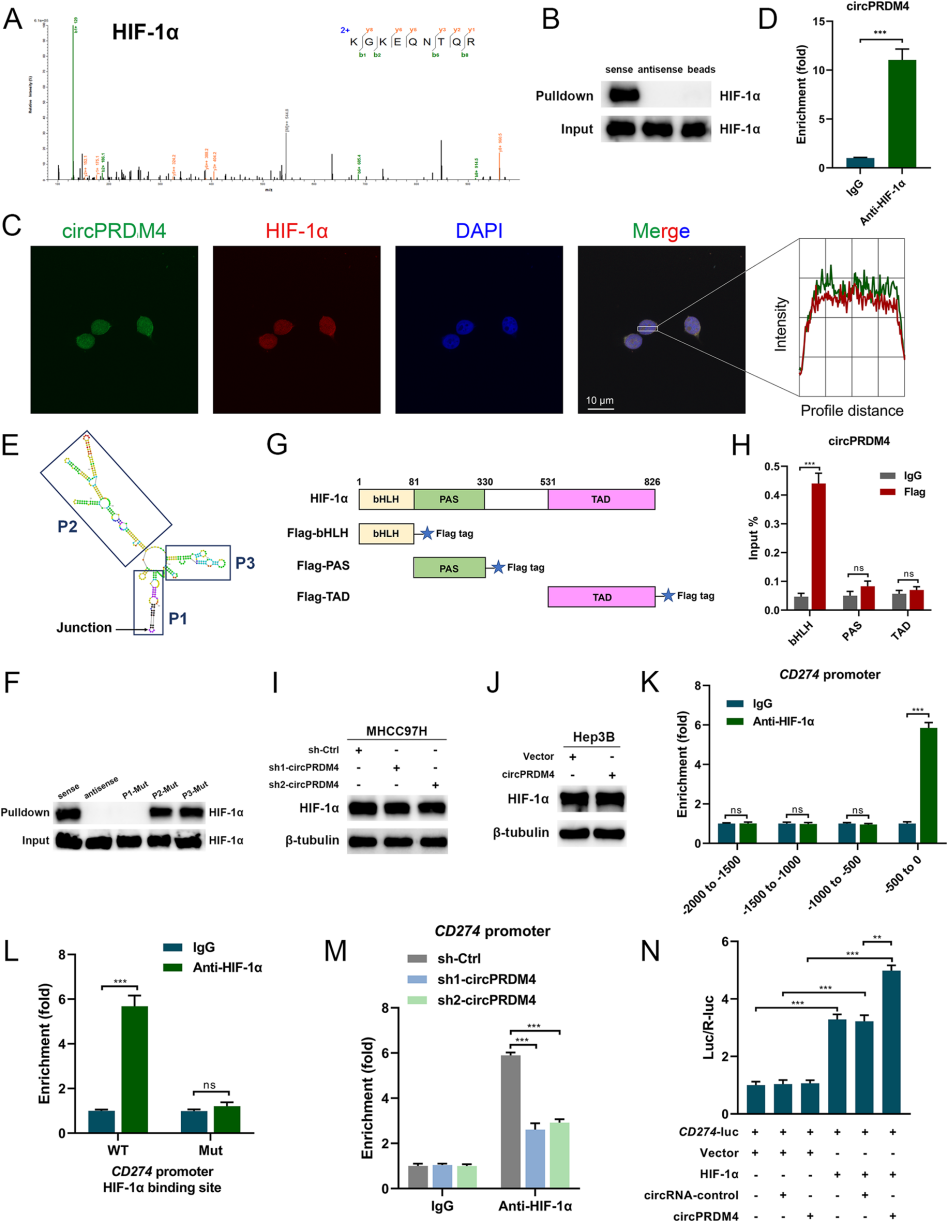
circPRDM4 recruits HIF-1α onto *CD274* promoter to facilitate HIF-1α-mediated transactivation of PD-L1. **A** Mass spectrum results indicating HIF-1α as a potential binding protein of circPRDM4 under hypoxia. **B** circRNA pull-down and western blotting assays confirming the interaction between circPRDM4 and HIF-1α under hypoxia. **C** Colocalization between circPRDM4 and HIF-1α under hypoxia. The profiles of colocalization are demonstrated. **D** RIP assays showing the binding between circPRDM4 and HIF-1α under hypoxia. **E** Secondary structure of circPRDM4 was established using RNAfold. **F** RNA pull-down assays were performed to show that the P1 fragment of circPRDM4 interacted with HIF-1α under hypoxia. **G** Schematic representation of HIF-1α functional domains and Flag-tagged HIF-1α variants are shown. **H** The binding between circPRDM4 and the bHLH domain of HIF-1α was examined after transfection of the Flag-tagged HIF-1α variants by RIP using anti-Flag antibody. IgG was used as a control. **I** Effects of circPRDM4 knockdown on HIF-1α protein expression levels under hypoxia. **J** Effects of circPRDM4 overexpression on HIF-1α protein expression levels under hypoxia. **K** ChIP assays were performed to confirm that HIF-1α could bind to *CD274* promoter (-500–0 bp). **L** ChIP assays showed the binding site of HIF-1α on *CD274* promoter (from -21 to -12 bp). **M** The binding between HIF-1α and *CD274* promoter was evaluated in circPRDM4-silenced cells using ChIP assays. **N** Dual-luciferase reporter assays showing that HIF-1α overexpression increased luciferase activities, while circPRDM4 overexpression alone exerted no significant effects on *CD274* transcription. Data are shown as mean ± SEM. **, *P* < 0.01; ***, *P* < 0.001; ns, no significance

To further understand the underlying mechanism of circPRDM4-mediated PD-L1 upregulation under hypoxic conditions, we took a circRNA pull-down approach to examining whether circPRDM4 could bind proteins. HCC cells were exposed to hypoxia treatment, and cell extracts were obtained. By incubating HCC cell extracts with a biotinylated probe complementary to circPRDM4, we enriched circPRDM4 and co-isolated a number of its potential binding proteins. Among the top candidates identified, HIF-1α most sparked our interest (Fig. 6A). Western blotting results confirmed the binding between circPRDM4 and endogenous HIF-1α under hypoxic conditions (Fig. 6B). In response to hypoxia treatment, circPRDM4 and HIF-1α colocalized intracellularly as shown in Fig. 6C. RIP assays confirmed the binding between circPRDM4 and HIF-1α (Fig. 6D).

To delineate the structural determinants for the interaction between circPRDM4 and HIF-1α, we first used RNAfold to construct the secondary structure of circPRDM4. Three well-characterized stem loop structures of circPRDM4 were predicted (Fig. 6E). RNA pull-down assays were performed with a series of circPRDM4 truncated fragments. As shown in Fig. 6F, the P1 fragment (nt 301–355 and 1–44) was found to be associated with HIF-1α. Next, we investigated the HIF-1α domain required for circPRDM4 binding by constructing Flag-tagged bHLH, PAS, and TAD of HIF-1α protein (Fig. 6G). We performed RIP assays and the results indicated that circPRDM4 bound to the bHLH domain but not to PAS nor TAD of HIF-1α (Fig. 6H). Together, we revealed that the P1 fragment of circPRDM4 bound to the bHLH domain of HIF-1α under hypoxia.

### circPRDM4 recruits HIF-1α onto *CD274* promoter to facilitate HIF-1α-mediated transactivation of PD-L1

Next, we wondered how the interaction between circPRDM4 and HIF-1α promoted PD-L1 expression. Based on the results from Fig. 6I, J and Additional file 5: Fig. S3A, B, circPRDM4 did not affect the protein, mRNA, or subcellular distribution of HIF-1α. Previous studies demonstrated that HIF-1α could transcriptionally activate PD-L1 [12, 13]. Notably, the DNA-binding ability of HIF-1α is mediated by its bHLH domain [20]. Therefore, we speculated that circPRDM4 might affect the HIF-1α-mediated transactivation of PD-L1. As shown in Fig. 6K, we performed ChIP assays and found that HIF-1α could bind to the promoter of *CD274* (− 500 to 0 bp). Previous report has identified the binding site of HIF-1α between -21 and -12 bp within *CD274* promoter [21]. As indicated in Fig. 6L, ChIP assays further confirmed the binding of HIF-1α to *CD274* promoter sequence (from − 21 to − 12 bp). circPRDM4 knockdown significantly suppressed the binding between HIF-1α and *CD274* promoter (Fig. 6M). Results from dual-luciferase reporter assays demonstrated that HIF-1α overexpression elevated the luciferase activities of *CD274* promoter compared to the negative control vector, whereas circPRDM4 overexpression alone exerted no significant effects on *CD274* transcription (Fig. 6N). In an effort to assess whether circPRDM4 could influence the recruitment of p300/CBP during HIF-1α binding to DNA, we performed IP of p300/CBP and HIF-1α in circPRDM4-overexpressing or control cells. No significant differences in terms of interaction between p300/CBP and HIF-1α were observed (Additional file 6: Fig. S4).

**Fig. 7:**
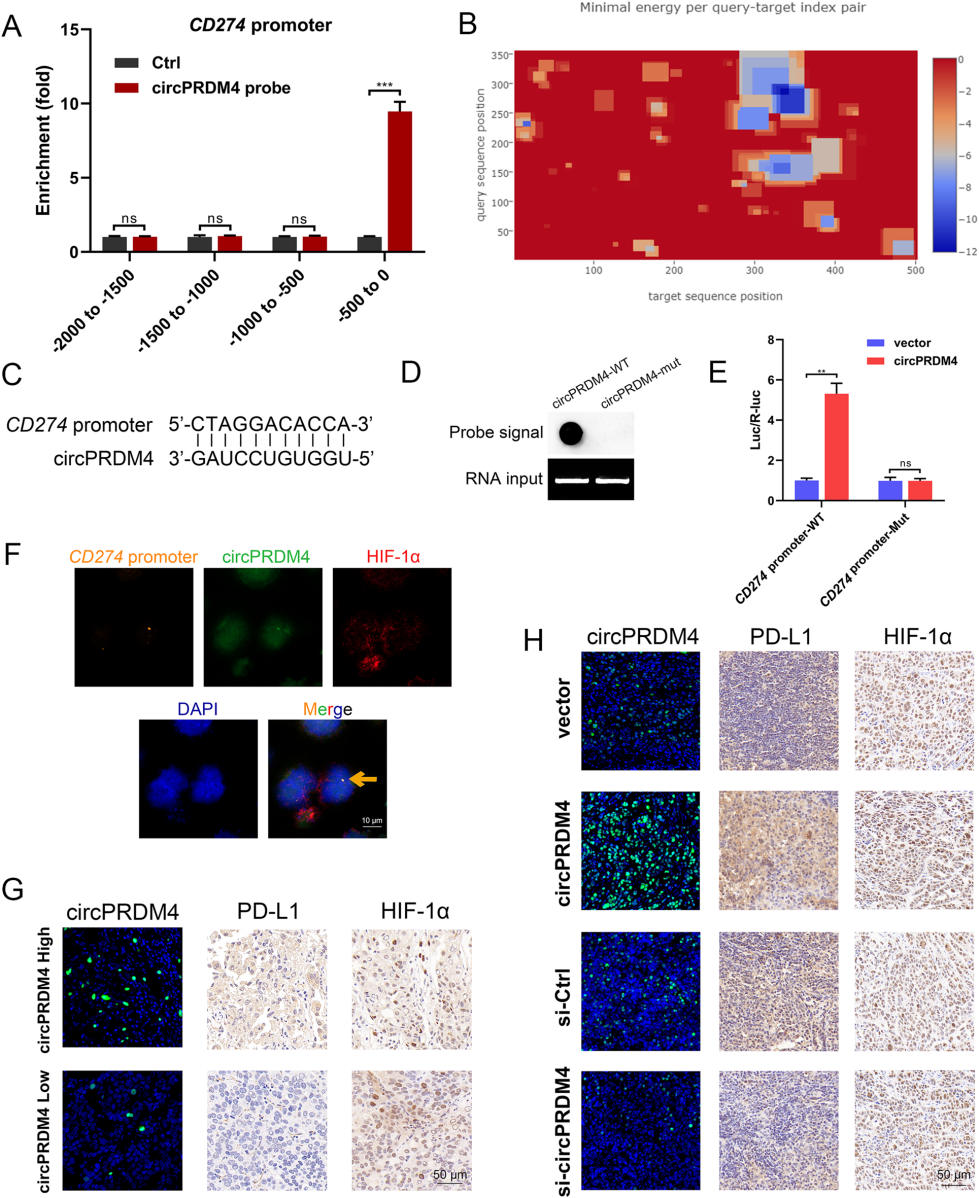
circPRDM4 binds to *CD274* promoter. **A** ChIRP assays were performed to determine the binding region between *CD274* promoter and circPRDM4. **B** Position-wise minimal energy profile that visualizes the minimal energy for each intermolecular index pair in a heatmap style. **C** Sequences of binding region between circPRDM4 transcript and *CD274* promoter. A base pairing between *CD274* promoter (from -170 to -160 bp) and circPRDM4 281–291 nt (within the P3 fragment) was predicted. **D** WT or mutant circPRDM4 transcripts were immobilized onto membrane, followed by probing with biotin-labeled single-strand DNA probes. **E** Luciferase reporter assay was performed in HIF-1α-overexpressing cells using indicated *CD274* promoters to validate circPRDM4 function on *CD274* transcription activation with the presence of HIF-1α. **F** DNA in situ hybridization.** G** Expression levels of HIF-1α, PD-L1, and circPRDM4 in HCC tissue sections of circPRDM4 high and circPRDM4 low groups. **H** Expression levels of HIF-1α, PD-L1, and circPRDM4 in PDX mouse models treated with adoptive human TIL-CD8^+^ T cell transfer. Data are shown as mean ± SEM. **, *P* < 0.01; ***, *P* < 0.001; ns, no significance

We then performed ChIRP assays and verified the specific binding between circPRDM4 and – 500 to 0 bp region of *CD274* promoter (Fig. 7A). The potential binding sites of circPRDM4 to *CD274* promoter were predicted by two bioinformatics software programs (IntaRNA and LncTar). A position-wise minimal energy profile that visualizes the minimal energy for each intermolecular index pair is provided in a heatmap style (Fig. 7B). The results of bioinformatics analysis indicated that the region of circPRDM4 from 281 to 291 nt may be a site with high affinity for the − 170 to − 160 bp region of *CD274* promoter (Fig. 7C). We generated the mutations of circPRDM4 transcript (circPRDM4-mut) to delete their base-pairing bases, followed by a hybridization assay. We found that circPRDM4-mut abolished the interaction between circPRDM4 transcript and *CD274* promoter (Fig. 7D). Substitution mutation in *CD274* promoter pairing region failed to activate luciferase activity by circPRDM4 overexpression in HIF-1α-overexpressing cells (Fig. 7E). In addition, DNA in situ hybridization showed that *CD274* promoter colocalized with circPRDM4 and HIF-1α (Fig. 7F). We applied the circPRDM4 truncated fragments to perform rescue experiments. We found that the binding between HIF-1α and *CD274* promoter (Additional file 7: Fig. S5A) and the luciferase activities of *CD274* promoter (Additional file 7: Fig. S5B) were significantly decreased in the P1-Mut group and the P3-Mut group, further confirming our findings. Moreover, we stained HIF-1α, PD-L1, and circPRDM4 in the tumor tissues of clinical samples and animal experiments. As shown in Fig. 7G, H, we found that tumor sections with higher circPRDM4 level exhibited higher PD-L1 expression level, whereas no significant difference in HIF-1α expression pattern was observed.

**Fig. 8:**
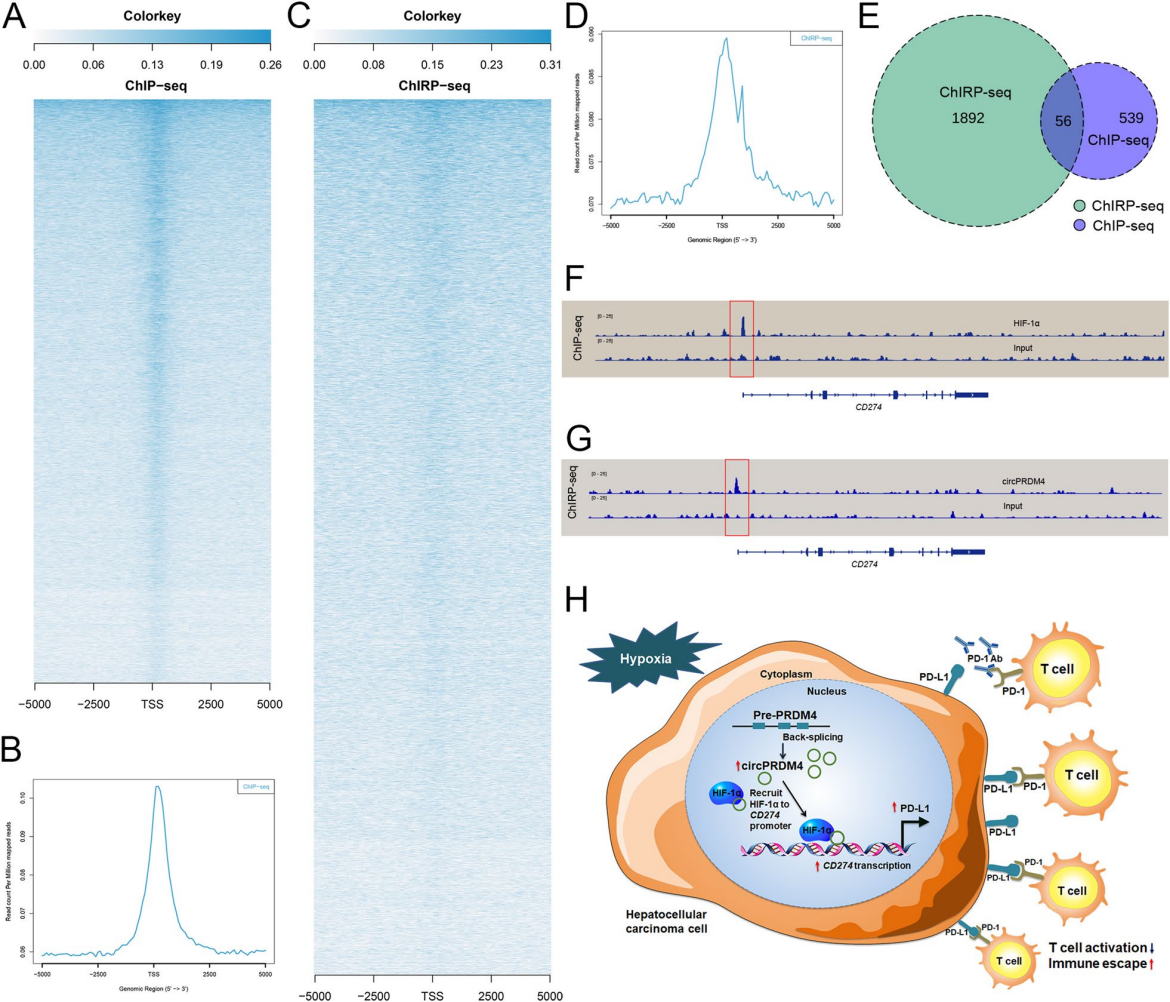
Genome-wide occupancy profiles of HIF-1α and circPRDM4. **A** Heatmap showing the binding sites of HIF-1α by ChIP-seq assay. **B** ChIP-seq analysis of HIF-1α genomic binding at target sites. A 10-kb interval centered on each HIF-1α peak is shown. **C** Heatmap showing the binding sites of circPRDM4 by ChIRP-seq assay. The signals near the peak center of circPRDM4-bound regions are plotted. **D** Global representation of circPRDM4 genomic binding over a 10-kb window centered on each circPRDM4 ChIRP-seq peak. **E** Venn graph showing the intersecting genes by ChIRP-seq assay for circPRDM4 and ChIP assay for HIF-1α. **F** ChIP-seq graph of *CD274* promoter bound by HIF-1α. **G** Representative ChIRP-seq of circPRDM4 binding of *CD274*. **H** Schematic diagram showing that circPRDM4 enhances HCC immune escape through HIF-1α regulation of PD-L1

We also assessed the effects of circPRDM4 overexpression on the expression levels of several HIF-1 target genes other than *CD274* (Additional file 8: Fig. S6). No significant increase in the expression levels of these genes was detected. To compare the genome-wide occupancy profiles of HIF-1α and circPRDM4, we performed ChIP-seq for HIF-1α and ChIRP-seq for circPRDM4 in HCCLM3 cells exposed to hypoxia treatment. The ChIP-seq signal of the binding sites of HIF-1α is shown in Fig. 8A. The signals near the peak center of each HIF-1α-bound region are plotted in Fig. 8B. For ChIRP-seq analysis, global visualization of circPRDM4 signals around gene transcriptional start site is demonstrated in Fig. 8C, 8D. A total of 56 genes were intersecting between HIF-1α ChIP-seq data and circPRDM4 ChIRP-seq data (Fig. 8E). As shown in Fig. 8F, HIF-1α was enriched in the promoter region of *CD274*. ChIRP-seq analysis demonstrated that circPRDM4 was enriched in *CD27* promoter region (Fig. 8G). These data reveal that HIF-1α and circPRDM4 can enrich in *CD274* promoter region in HCC.

In addition, we silenced HIF-1α or PD-L1 in circPRDM4-overexpressing HCC cells. As shown in Additional file 9: Fig. S7A, B, HIF-1α knockdown led to a decreased level of *CD274* in HCC cells overexpressing circPRDM4. According to the results from T cell cytotoxicity assays based on LDH release, HIF-1α knockdown compromised the immune evasion of circPRDM4-overexpressing cells (Additional file 9: Fig. S7C). These results indicated that circPRDM4 promoted PD-L1 transcription and HCC immune escape mainly through HIF-1α. Moreover, PD-L1 depletion hindered the immune escape of HCC cells caused by circPRDM4 overexpression (Additional file 10: Fig. S8A, B).

Taken together, these data indicated that circPRDM4 promoted HCC immune escape mainly through the HIF-1α/PD-L1 axis. A schematic model of circPRDM4 in immune escape of HCC cells under hypoxia is shown in Fig. 8H.

## Discussion

Blockade of immune checkpoints contributes to HCC regression, however, the response rate remains quite low [22]. Understanding the mechanisms underlying immune escape is necessary to achieve better survival outcomes in advanced HCC patients in the clinical context [23]. In this study, we found that circPRDM4 was highly expressed in responders for anti-PD-1 therapy. circPRDM4 expression level was correlated to the therapeutic efficacy of PD-1 blockade. Improved PFS and OS were observed in PD-1 blockade-treated HCC patients with high circPRDM4 expression. In addition, circPRDM4 upregulation was associated with less CD8^+^ cell infiltration and high *CD274* expression level. *CD274* mRNA regulation is an intricate process, which involves genomic alterations, epigenetic modification, transcriptional regulation, and post-transcriptional modification [4]. Future studies with larger sample size are warranted to confirm the correlation between circPRDM4 and *CD274* mRNA level and to piece together the puzzle of *CD274* regulation network.

Hypoxia has been acknowledged as a potent factor that impedes anti-tumor immunity by reshaping the tumor microenvironment [24]. HIF-1α is a core regulator under hypoxic conditions, and has been proved as a direct regulator of PD-L1 [12, 13]. We performed ChIP and dual-luciferase reporter assays and confirmed that HIF-1α bound to *CD274* promoter and transactivated PD-L1. Therefore, tumor cells may leverage the HIF-1α/PD-L1 axis to evade from immune surveillance. Reportedly, SLC7A11 upregulates PD-L1 expression through the HIF-1α cascade and contributes to the formation of an immunosuppressive microenvironment, thus promoting HCC metastasis [21]. In glioma cells, the direct binding between HIF-1α and *CD274* promoter region results in elevated PD-L1 expression under hypoxia [25]. Here, we indicated that circPRDM4 expression level was increased under hypoxia conditions in HCC cells. Since expression of PD-L1 is frequently detected in human malignancies, PD-L1 expression on cancer cells and other cells in the tumor microenvironment is of major clinical relevance [26]. In the present study, we focused on the effects of circPRDM4 on PD-L1 expression in HCC cells. We performed loss- and gain-of function experiments, and showed that circPRDM4 inhibited CD8^+^ T cell-mediated anti-tumor immunity mainly through upregulating PD-L1. Results from RNA pull-down, mass spectrometry, and RIP assays indicated that circPRDM4 could interact with HIF-1α. Rescue experiments verified that circPRDM4 facilitated immune escape of HCC cells mainly by the HIF-1α/PD-L1 axis.

Mechanistically, our work revealed that circPRDM4 not only bound to the bHLH domain of HIF-1α but also the promoter of *CD274*. The bHLH domain defines a large superfamily of eukaryotic transcription factors and mediates the combination of HIF-1α and DNA [20]. We therefore investigated the role of circPRDM4 in the interaction between HIF-1α and *CD274* promoter. Intriguingly, we found that circPRDM4 could function as a scaffold to recruit HIF-1α onto *CD274* promoter, and cemented their interaction, ultimately promoting the HIF-1α-mediated transactivation of PD-L1. Knockdown of circPRDM4 impeded the interaction between HIF-1α and *CD274* promoter, and dampened the expression level of PD-L1. Recently, emerging evidence has revealed that circRNAs can serve as scaffold to recruit proteins, especially transcription factors, to chromatin [27]. circIPO11 recruits TOP1 to *GLI1* promoter to activate its transcription, and drives self-renewal of liver cancer initiating cells [28]. circKcnt2 recruits the NuRD complex to inhibit *Batf* transcription, thus facilitating colitis resolution [29]. In prostate cancer, circ0005276 interacts with FUS so as to initiate the transcription of *XIAP* [30]. Upregulated circAnks1a in spinal cord increases YBX1 recruitment to *Vegfb* promoter, thereby triggering its transcription [31]. In HCC, circRHOT1 recruits TIP60 to the promoter region of *NR2F6* and facilitates its transcription [32]. circPOK interacts with ILF2/3 complex to promote *Il6* transcription in mesenchymal tumors [33]. Our work demonstrated that the circPRDM4-HIF-1α-*CD274* ternary complex reinforced the interaction of HIF-1α with *CD274* promoter. This interaction was at least in part required for the tumor-promoting roles of circPRDM4 in immune evasion of HCC cells under hypoxic conditions.

As reported by various clinical trials, among the patients treated with anti-PD-1 monotherapy, PD-L1 positive HCCs respond better than those with negative PD-L1 expression [34–36]. Genomic analyses for the GO30140 phase Ib and IMbrave150 phase 3 trial of atezolizumab and bevacizumab in patients with HCC reported that high expression of PD-L1, as per RNA-seq, is related to better response and longer PFS [37]. A preclinical study by Liu et al*.* reported that ADORA1 expression is negatively correlated with PD-L1 expression, and patients with low ADORA1 expression show tumor shrinkage after PD-1 mAb treatment [38]. In the present study, we found that circPRDM4 induced PD-L1 expression, and circPRDM4 was related to better response to PD-1 blockade.

In conclusion, hypoxia-associated circPRDM4 promoted PD-L1 transcription and contributed to HCC cell evading from CD8^+^ T cell-mediated anti-tumor immunity. To our knowledge, the present work reveals the first evidence showing a circRNA acting as a scaffold in HIF-1α-mediated immune escape. The roles of circPRDM4 in hypoxic immunosuppressive microenvironment not only present novel insight into immune escape of tumor cells, but also provide a novel prognostic biomarker and therapeutic candidate for cancer immunotherapy.

## Supplementary Information

**Additional file 1: Table S1.** Primers for quantitative real-time PCR used in this study.

**Additional file 2.** Supplementary materials and methods.

**Additional file 3: Fig. S1.** circPRDM4 does not affect HCC cell survival or growth in the absence of immune cells. A Effects of circPRDM4 knockdown on MHCC97H and HCCLM3 cell survival or growth in the absence of immune cells. B Effects of circPRDM4 overexpression on Hep3B and HepG2 cell survival or growth in the absence of immune cells. C Representative tumor images of each group of immunodeficient mice at the end of treatment. D Tumor weights of the xenografts with circPRDM4 overexpression under immunodeficient conditions at the end of treatment. E Tumor weights of the xenografts with circPRDM4 knockdown under immunodeficient conditions at the end of treatment. F Tumor growth curves of the xenografts with circPRDM4 overexpression under immunodeficient conditions. G Tumor growth curves of the xenografts with circPRDM4 knockdown under immunodeficient conditions. H Expression levels of circPRDM4 in xenografts with circPRDM4 overexpression under immunodeficient conditions. I Expression levels of circPRDM4 in xenografts with circPRDM4 knockdown under immunodeficient conditions. Data are shown as mean ± SEM. ***, P < 0.001; ns, no significance.

**Additional file 4: Fig. S2.** circPRDM4 has no influence on its host gene and lacks protein-coding potential. A Western blotting results showing the impact of circPRDM4 knockdown on PRDM4 protein expression levels. B CPAT results indicating no protein-coding potential of circPRDM4. C circRNADb results suggesting that circPRDM4 lacks protein-coding potential.

**Additional file 5: Fig. S3.** circPRDM4 did not affect HIF1A mRNA levels and HIF-1α subcellular distribution. A Effects of circPRDM4 knockdown on HIF1A mRNA expression levels under hypoxia. B Subcellular distribution of HIF-1α upon circPRDM4 knockdown under hypoxia. Data are shown as mean ± SEM. ns, no significance.

**Additional file 6: Fig. S4.** IP assays to measure the interactions between p300/CBP and HIF-1α in circPRDM4-overexpressing and control cells.

**Additional file 7: Fig. S5.** Effects of circPRDM4 truncated mutants in CD274 transcription activity. A ChIP assays were performed to assess the binding between HIF-1α and CD274 promoter in circPRDM4 truncated mutants. B Dual-luciferase reporter assays were used to evaluate the luciferase activities in circPRDM4 truncated mutants. Data are shown as mean ± SEM. **, P < 0.01; ns, no significance.

**Additional file 8: Fig. S6.** Effects of circPRDM4 overexpression on the expression levels of several HIF-1 target genes, including VEGF, LDHA, PDK1, and GLUT1. HIF-2 target CITED2 was used as a control. Data are shown as mean ± SEM. ns, no significance.

**Additional file 9: Fig. S7.** circPRDM4 promotes HCC immune escape mainly through HIF-1α. A Expression levels of HIF1A mRNA in circPRDM4-overexpressing HCC cells with or without HIF-1α knockdown. B Expression levels of CD274 mRNA in circPRDM4-overexpressing HCC cells with or without HIF-1α knockdown. C LDH release assay results showing the cytotoxicity in circPRDM4-overexpressing HCC cells with or without HIF-1α knockdown. Data are shown as mean ± SEM. **, P < 0.01; ***, P < 0.001; ns, no significance.

**Additional file 10: Fig. S8.** circPRDM4 facilitates HCC immune escape mainly by PD-L1. A Expression levels of CD274 mRNA in circPRDM4-overexpressing HCC cells with or without PD-L1 knockdown. B LDH release assay results showing the cytotoxicity in circPRDM4-overexpressing HCC cells with or without PD-L1 knockdown. Data are shown as mean ± SEM. **, P < 0.01; ***, P < 0.001.

## Data Availability

The datasets used and/or analyzed during the current study are available from the corresponding authors on reasonable request.

## Abbreviations

HCC: Hepatocellular carcinoma
PD-1: Programmed death 1
PD-L1: Programmed death-ligand 1
circRNA: Circular RNA
RT-qPCR: Quantitative real-time PCR
FISH: Fluorescence in situ hybridization
DAPI: 4',6-Diamidino-2-phenylindole
LDH: Lactate dehydrogenase
ELISA: Enzyme linked immunosorbent assay
PDX: Patient-derived xenograft
TIL: Tumor infiltrating lymphocyte
RIP: RNA immunoprecipitation
bHLH: Basic helix-loop-helix
PAS: Per-ARNT-Sim
TAD: Transactivation domain
ChIP: Chromatin immunoprecipitation
ChIRP: Chromatin isolation by RNA purification
IP: Immunoprecipitation
PFS: Progression-free survival
OS: Overall survival
shRNA: Short hairpin RNA
