# Supplementary material for: Hypoxia-associated circPRDM4 promotes immune escape via HIF-1α regulation of PD-L1 in hepatocellular carcinoma
Source: Exp Hematol Oncol. 2023 Feb 6;12:17. doi: 10.1186/s40164-023-00378-2 (PMC9903500; doi:10.1186/s40164-023-00378-2)
Supplement: Supplementary file 1 — Additional file 1: Table S1. Primers for quantitative real-time PCR used in this study. [file 40164_2023_378_MOESM1_ESM.docx]

**Table S1**. Primers for quantitative real-time PCR used in this study

| Genes | Forward primer | Reverse primer |
| --- | --- | --- |
| hsa_circ_0000615 | CAGCGCTCAATCCTTTGGGA | GACCTGCCACATTGGTCAGTA |
| hsa_circ_0007468 | GGACAGACAAGGCAGTTAACCATAT | GGTCACACAGAGTACACCCTGG |
| hsa_circ_0138414 | GGGGCAGCAGTATTGTGAAA | AAGACTGTGTGCTCCCCATT |
| hsa_circ_0032138 | AGGACAGTACAGGATGCTTGCC | ATATCCCATCAATTCGGTAATTCTC |
| hsa_circ_0008450 | TGATGCATCCCATCCACCCT | AGCCCTGCCACTTGTCATTC |
| PRDM4 | CCGGTCGACGAAAACATGCATCACAGGATG | CGCGGATCCGTTATTTATGTGCAGAAAGA |
| CD274 | GCTGCACTAATTGTCTATTGGGA | AATTCGCTTGTAGTCGGCACC |
| HIF1A | TCCTTCGGACACATAAGCTCC | GACAGAAAGATCATGTCACCGT |
| VEGFA | AGGGCAGAATCATCACGAAGT | AGGGTCTCGATTGGATGGCA |
| LDHA | ATGGCAACTCTAAAGGATCAGC | CCAACCCCAACAACTGTAATCT |
| PDK1 | CTGTGATACGGATCAGAAACCG | TCCACCAAACAATAAAGAGTGCT |
| GLUT1 | GGCCAAGAGTGTGCTAAAGAA | ACAGCGTTGATGCCAGACAG |
| CITED2 | CCTAATGGGCGAGCACATACA | GGGGTAGGGGTGATGGTTGA |
| GAPDH | GCATTGCCCTCAACGACCAC | CCACCACCCTGTTGCTGTAG |
| U6 | CTCGCTTCGGCAGCACA | AACGCTTCACGAATTTGCGT |
| β-actin | TCACCAACTGGGACGACATG | GTCACCGGAGTCCATCACGAT |
