# Supplementary material for: Hypoxia-associated circPRDM4 promotes immune escape via HIF-1α regulation of PD-L1 in hepatocellular carcinoma
Source: Exp Hematol Oncol. 2023 Feb 6;12:17. doi: 10.1186/s40164-023-00378-2 (PMC9903500; doi:10.1186/s40164-023-00378-2)
Supplement: Supplementary file 2 — Additional file 2. Supplementary materials and methods. [file 40164_2023_378_MOESM2_ESM.docx]

**Supplementary Materials and Methods**

**Cell lines and cell culture**

HCCLM3, Hep3B, HepG2, Huh7, MHCC97L, Focus, and MHCC97H cell lines were obtained from the Key Laboratory of Liver Transplantation, Chinese Academy of Medical Sciences (Nanjing, China). All cells were cultured in DMEM (Gibco, Carlsbad, CA, USA) supplemented with 10% fetal bovine serum (Gibco) and 1% penicillin/streptomycin (Gibco).

**RNA stability assay**

For actinomycin D treatment, MHCC97H and Hep3B cells were exposed to 2 μg/mL of actinomycin D (Sigma-Aldrich, St. Louis, MO, USA) for 0 h, 6 h, 12 h, and 24 h, respectively. Total RNA was isolated, and RT-qPCR was performed to quantify the relative levels of circPRDM4 and *PRDM4* mRNA.

**Patient samples**

A total of 20 HCC patients with recurrence or distant metastasis who received Camrelizumab (an anti-PD-1 mAb, Jiangsu Hengrui Pharmaceuticals Co., Ltd., China) monotherapy at the First Affiliated Hospital of Wannan Medical College were included in this study. The diagnosis of HCC was determined by two independent pathologists based on histological examination. The enrolled patients had a performance status defined by the Eastern Cooperative Oncology Group of 0 or 1. Archival tumor tissues from prior surgery with curative intent or excisional biopsy before the start of anti-PD-1 therapy were obtained. This study was approved by the Ethics Committee of the First Affiliated Hospital of Wannan Medical College and the Ethics Committee of the First Affiliated Hospital of Nanjing Medical University. The study protocol conformed to the provisions of the Declaration of Helsinki. Written informed consent was obtained from all patients.

**Lentiviral infection and plasmid transfection**

Short hairpin RNAs (shRNAs) targeting circPRDM4 and HIF-1α, lentiviruses overexpressing circPRDM4, and small interfering RNAs (siRNAs) targeting circPRDM4 and PD-L1 were designed and purchased from GenePharma (Shanghai, China). After lentiviral transfection, cells were selected by 1 μg/mL of puromycin treatment. For the plasmid and siRNA transfection, we used Lipofectamine 3000 (Invitrogen, Carlsbad, CA, USA) based on the manufacturer’s protocols. The sequences used in this study were as follows: sh1-circPRDM4: 5′-GTGCGCAAAGCCAGGGTGT-3′, sh2-circPRDM4: 5′-GCAAAGCCAGGGTGTACTC-3′; sh-HIF-1α: 5’-CTGATGACCAGCAACTTGA-3’; si-circPRDM4: 5′-GUGCGCAAAGCCAGGGUGU-3′; si-PD-L1: 5’-CAAAAUCAACCAAAGAAUU-3’.

**Western blotting**

Cells were dissolved in RIPA and the protein concentrations were determined using a BCA Protein Assay Kit (Beyotime, Shanghai, China). Proteins were separated using SDS-PAGE and then transferred to PVDF membranes (EMD Millipore, Billerica, MA, USA). The membranes were blocked with 5% bovine serum albumin in TBST and then incubated with the primary antibodies at 4℃ overnight. The next day, membranes were washed in TBST three times for 15 min, and incubated with secondary antibodies for 2 h at room temperature. Super ECL Detection Reagent (Yeasen, Shanghai, China) was used to detect the immunocomplexes. Image Lab (Bio-Rad, Hercules, CA, USA) was used for visualization. The dilutions for specific antibodies were as follows: 1:1000 for PD-L1 (Cell Signaling Technology, Danvers, MA, USA), 1:1000 for HIF-1α (Cell Signaling Technology), 1:1000 for PRDM4 (Abcam, Waltham, MA, USA), 1:1000 for β-tubulin (Cell Signaling Technology), 1:2000 for histone H3 (Cell Signaling Technology).

**Immunohistochemistry (IHC)**

Tissue specimens were fixed in 4% paraformaldehyde and embedded in paraffin sections. The primary antibodies against PD-L1 (1:100 dilution; Cell Signaling Technology) and HIF-1α (1:100 dilution; Abcam) were used for IHC staining. After incubation with the primary antibodies at 4℃ overnight, the sections were subjected to horseradish peroxidase-conjugated secondary antibodies and horseradish peroxidase substrate 3,3'-diaminobenzidine. The sections were scanned under a microscope (Nikon Corporation, Tokyo, Japan).
