# Supplementary figures and images for: Hypoxia-associated circPRDM4 promotes immune escape via HIF-1α regulation of PD-L1 in hepatocellular carcinoma
Source: Exp Hematol Oncol. 2023 Feb 6;12:17. doi: 10.1186/s40164-023-00378-2 (PMC9903500; doi:10.1186/s40164-023-00378-2)

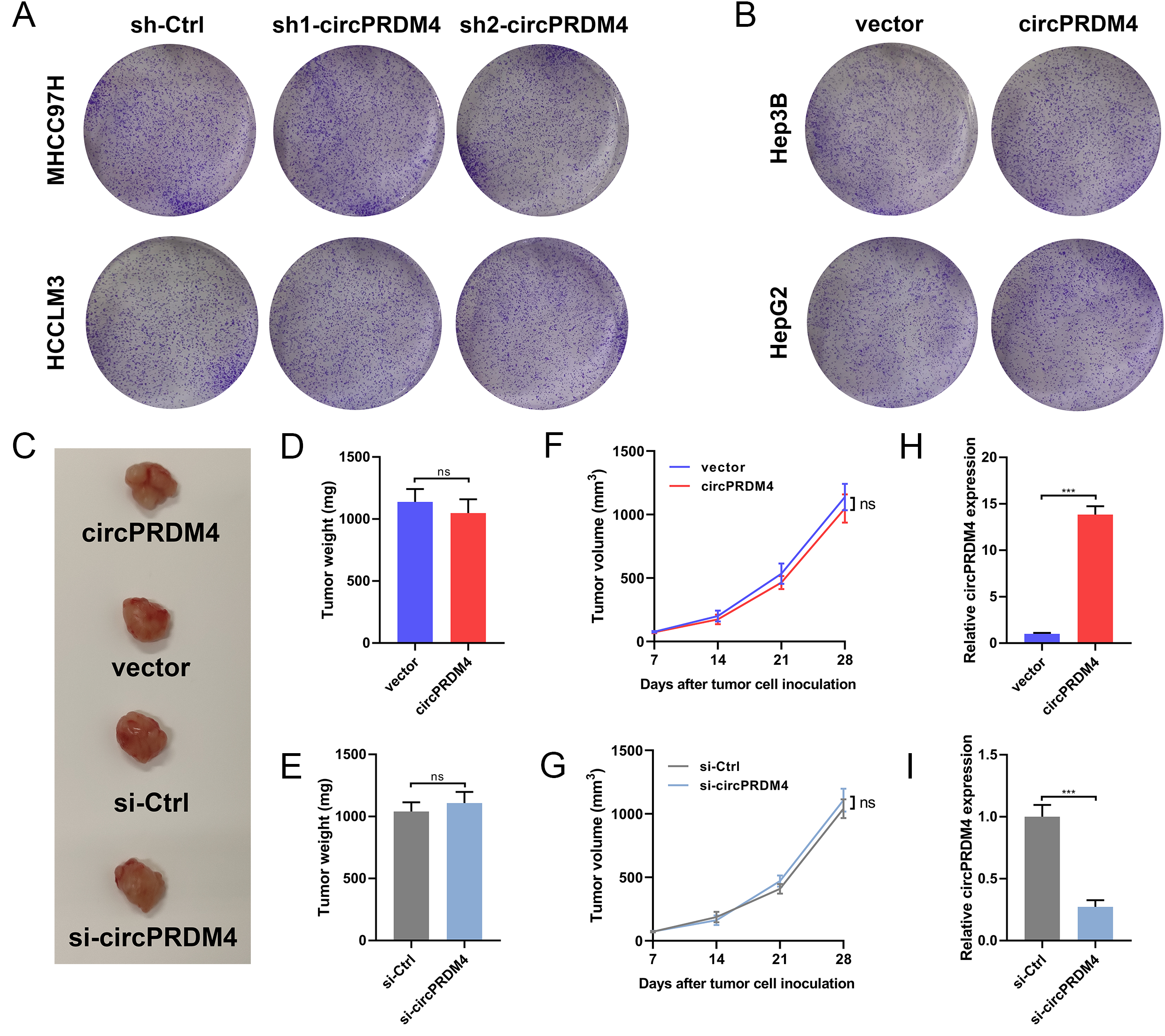

Supplement: Supplementary file 3 — Additional file 3: Fig. S1. circPRDM4 does not affect HCC cell survival or growth in the absence of immune cells. A Effects of circPRDM4 knockdown on MHCC97H and HCCLM3 cell survival or growth in the absence of immune cells. B Effects of circPRDM4 overexpression on Hep3B and HepG2 cell survival or growth in the absence of immune cells. C Representative tumor images of each group of immunodeficient mice at the end of treatment. D Tumor weights of the xenografts with circPRDM4 overexpression under immunodeficient conditions at the end of treatment. E Tumor weights of the xenografts with circPRDM4 knockdown under immunodeficient conditions at the end of treatment. F Tumor growth curves of the xenografts with circPRDM4 overexpression under immunodeficient conditions. G Tumor growth curves of the xenografts with circPRDM4 knockdown under immunodeficient conditions. H Expression levels of circPRDM4 in xenografts with circPRDM4 overexpression under immunodeficient conditions. I Expression levels of circPRDM4 in xenografts with circPRDM4 knockdown under immunodeficient conditions. Data are shown as mean ± SEM. ***, P < 0.001; ns, no significance. [file 40164_2023_378_MOESM3_ESM.tif]

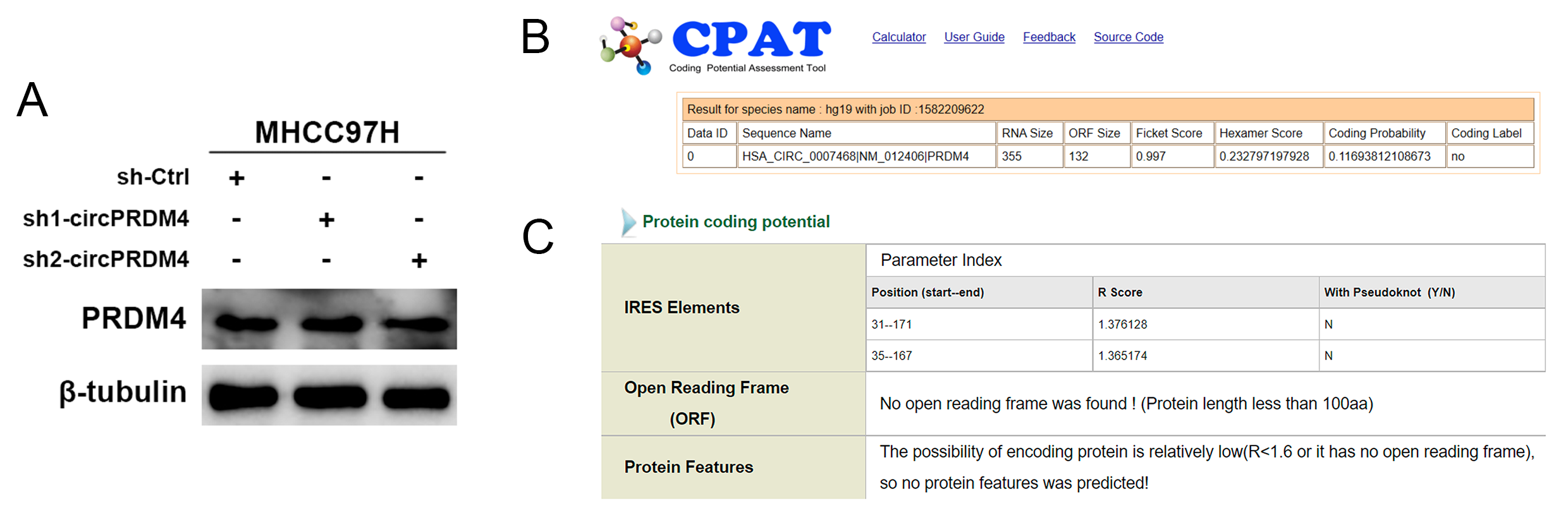

Supplement: Supplementary file 4 — Additional file 4: Fig. S2. circPRDM4 has no influence on its host gene and lacks protein-coding potential. A Western blotting results showing the impact of circPRDM4 knockdown on PRDM4 protein expression levels. B CPAT results indicating no protein-coding potential of circPRDM4. C circRNADb results suggesting that circPRDM4 lacks protein-coding potential. [file 40164_2023_378_MOESM4_ESM.tif]

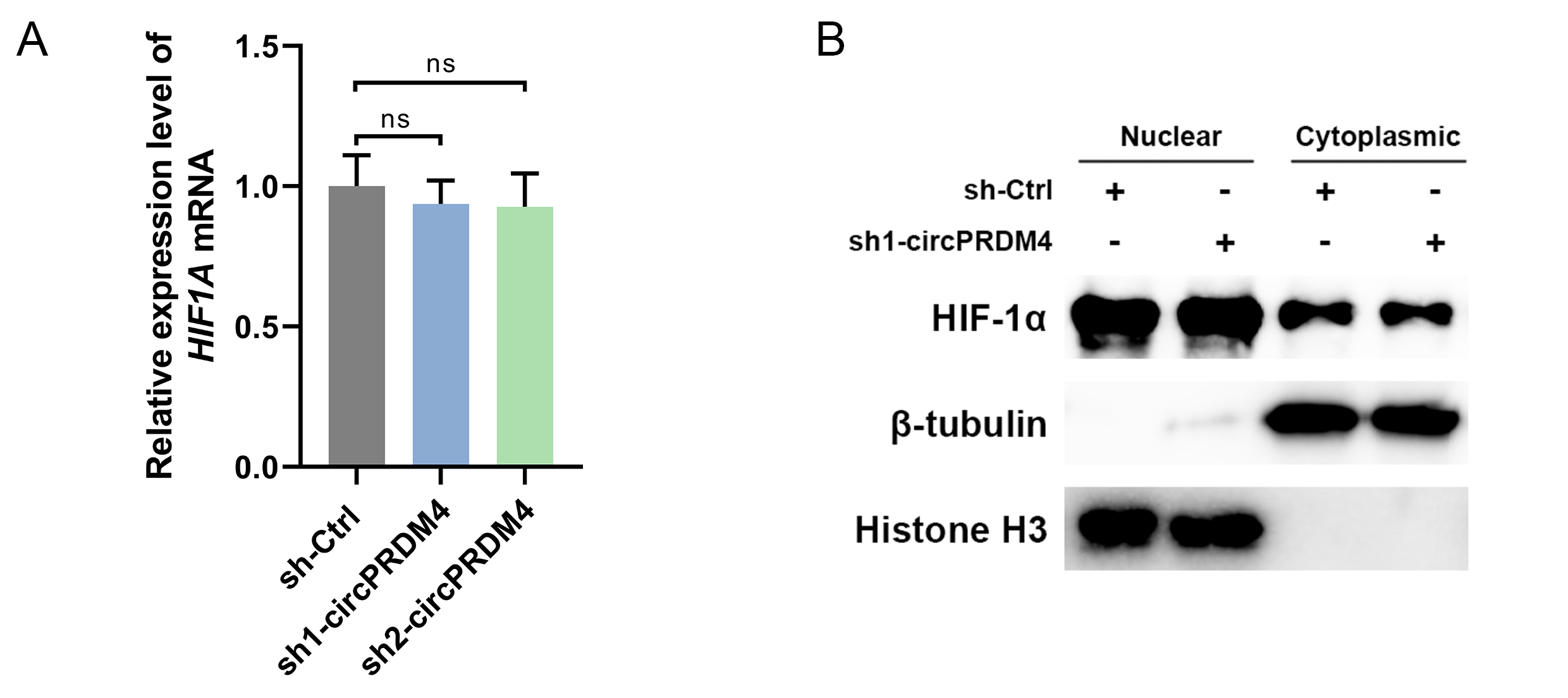

Supplement: Supplementary file 5 — Additional file 5: Fig. S3. circPRDM4 did not affect HIF1A mRNA levels and HIF-1α subcellular distribution. A Effects of circPRDM4 knockdown on HIF1A mRNA expression levels under hypoxia. B Subcellular distribution of HIF-1α upon circPRDM4 knockdown under hypoxia. Data are shown as mean ± SEM. ns, no significance. [file 40164_2023_378_MOESM5_ESM.tif]

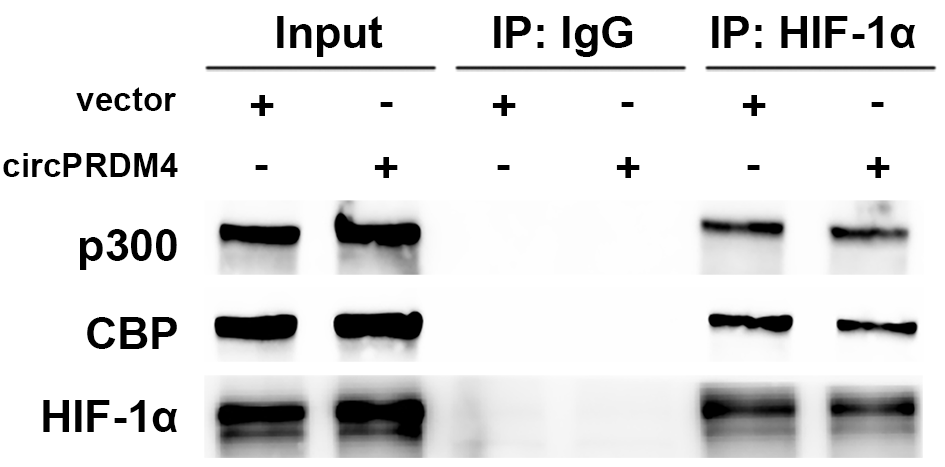

Supplement: Supplementary file 6 — Additional file 6: Fig. S4. IP assays to measure the interactions between p300/CBP and HIF-1α in circPRDM4-overexpressing and control cells. [file 40164_2023_378_MOESM6_ESM.tif]

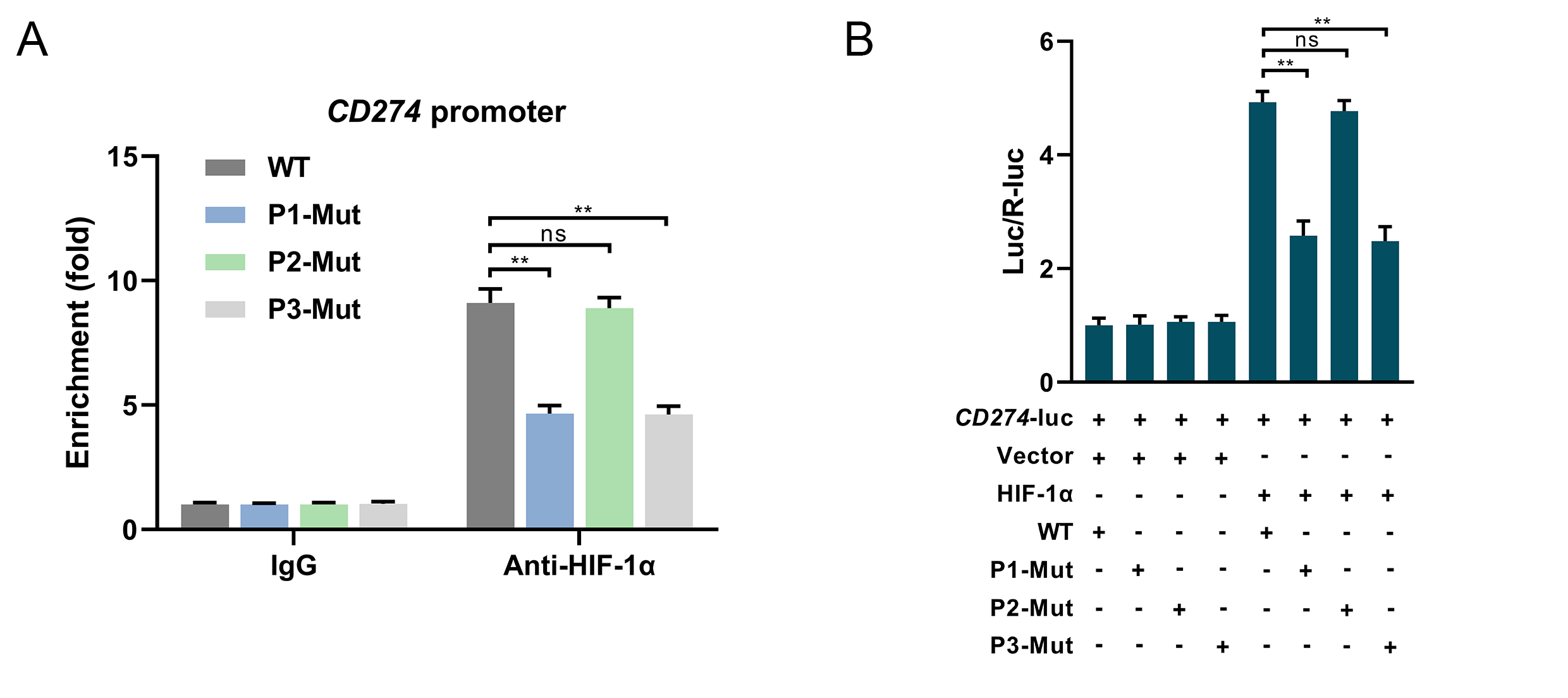

Supplement: Supplementary file 7 — Additional file 7: Fig. S5. Effects of circPRDM4 truncated mutants in CD274 transcription activity. A ChIP assays were performed to assess the binding between HIF-1α and CD274 promoter in circPRDM4 truncated mutants. B Dual-luciferase reporter assays were used to evaluate the luciferase activities in circPRDM4 truncated mutants. Data are shown as mean ± SEM. **, P < 0.01; ns, no significance. [file 40164_2023_378_MOESM7_ESM.tif]

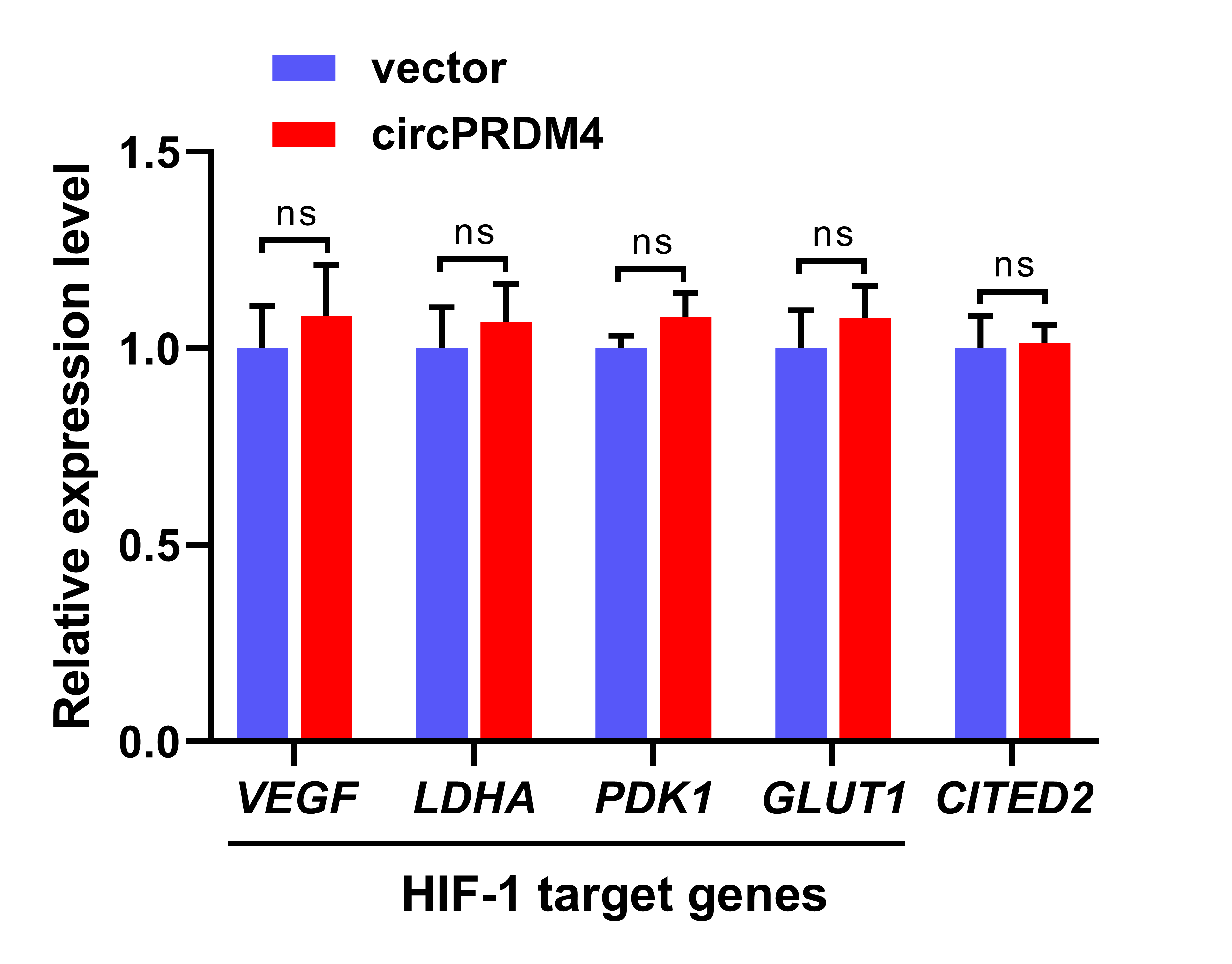

Supplement: Supplementary file 8 — Additional file 8: Fig. S6. Effects of circPRDM4 overexpression on the expression levels of several HIF-1 target genes, including VEGF, LDHA, PDK1, and GLUT1. HIF-2 target CITED2 was used as a control. Data are shown as mean ± SEM. ns, no significance. [file 40164_2023_378_MOESM8_ESM.tif]

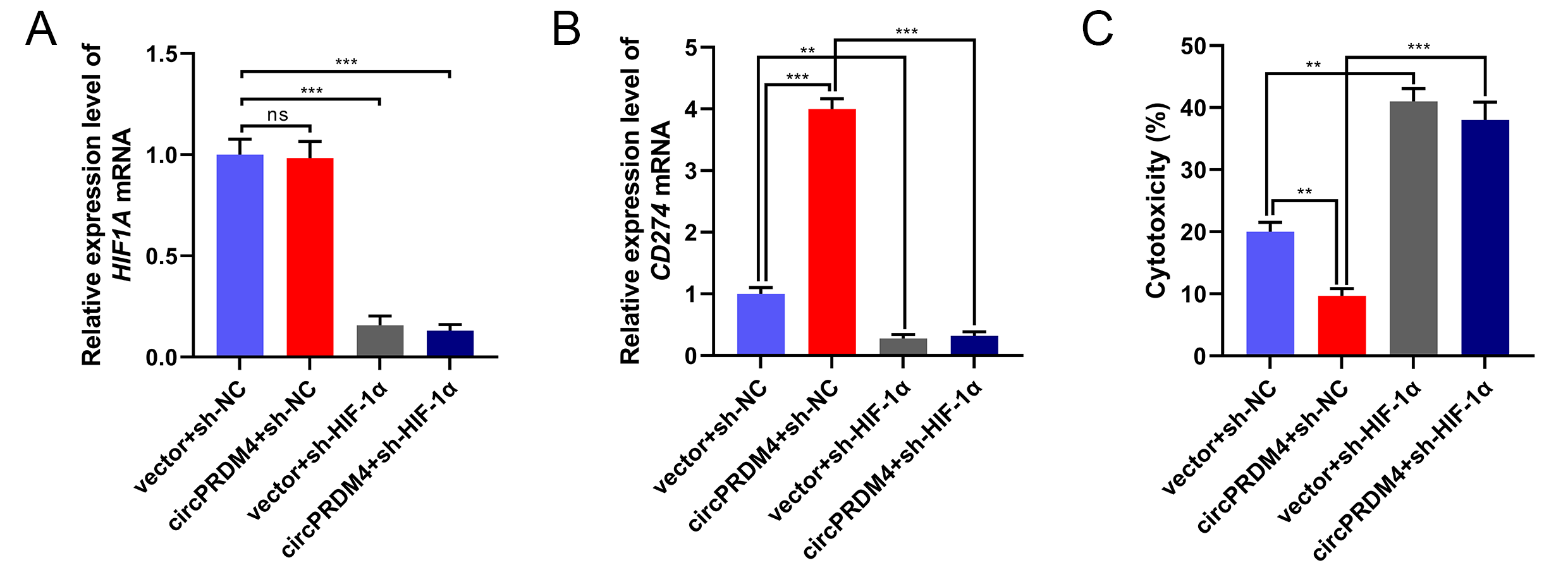

Supplement: Supplementary file 9 — Additional file 9: Fig. S7. circPRDM4 promotes HCC immune escape mainly through HIF-1α. A Expression levels of HIF1A mRNA in circPRDM4-overexpressing HCC cells with or without HIF-1α knockdown. B Expression levels of CD274 mRNA in circPRDM4-overexpressing HCC cells with or without HIF-1α knockdown. C LDH release assay results showing the cytotoxicity in circPRDM4-overexpressing HCC cells with or without HIF-1α knockdown. Data are shown as mean ± SEM. **, P < 0.01; ***, P < 0.001; ns, no significance. [file 40164_2023_378_MOESM9_ESM.tif]

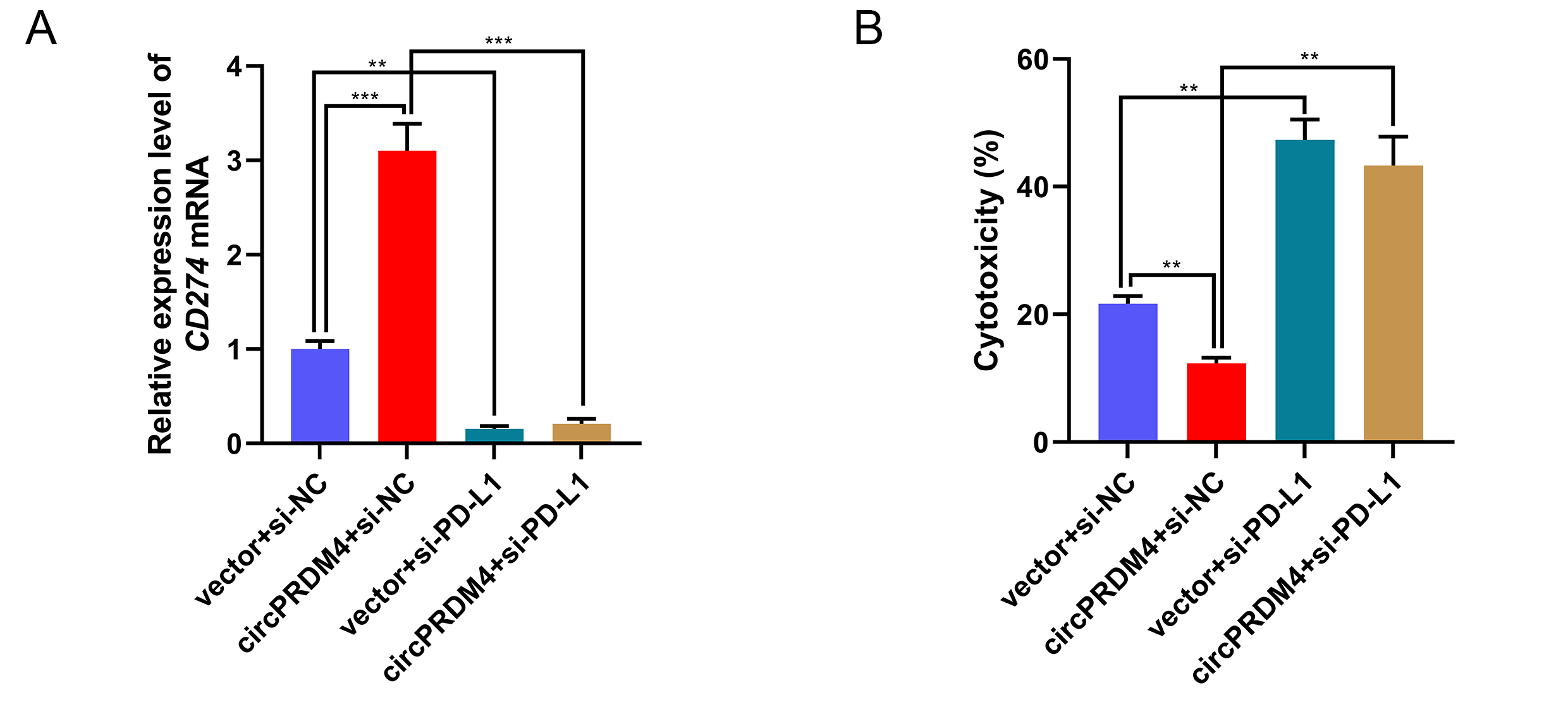

Supplement: Supplementary file 10 — Additional file 10: Fig. S8. circPRDM4 facilitates HCC immune escape mainly by PD-L1. A Expression levels of CD274 mRNA in circPRDM4-overexpressing HCC cells with or without PD-L1 knockdown. B LDH release assay results showing the cytotoxicity in circPRDM4-overexpressing HCC cells with or without PD-L1 knockdown. Data are shown as mean ± SEM. **, P < 0.01; ***, P < 0.001. [file 40164_2023_378_MOESM10_ESM.tif]
